# Association of Neurotensin Receptor 1 Gene Polymorphisms With Defense Mechanisms in Healthy Chinese

**DOI:** 10.3389/fpsyt.2021.762276

**Published:** 2021-11-17

**Authors:** Hui Ma, Min Li, Liguo Zhang, Jiangang Tao, Gang Zhu

**Affiliations:** ^1^Center for Mental Health, Yanshan University, Qinhuangdao, China; ^2^Department of Psychiatry, The First Affiliated Hospital of China Medical University, Shenyang, China; ^3^Center for Mental Health, Hebei Institute of International Business and Economics, Qinhuangdao, China; ^4^Department of Psychiatry, The Third Hospital of Heilongjiang Province, Bei'an, China

**Keywords:** defense mechanism, defense style questionnaire, neurotensin receptor, gene polymorphism, Chinese

## Abstract

**Aims:** In the central nerve system, neurotensin (NT), and neurotensin receptor 1 (NTR1) modulate the dopamine system. Gene variations in the dopamine system have been demonstrated to influence certain defense mechanisms, but no studies have investigated possible effect of *NTR1* gene polymorphisms in the biological determination of these defenses. The present study therefore examined this link.

**Methods:** In 412 healthy Han Chinese, single nucleotide polymorphisms rs6090453C/G, rs6011914C/G, and rs2427422A/G of the *NTR1* gene were genotyped, and the defense mechanisms were measured by the self-reporting Defense Style Questionnaire 88.

**Results:** Significant male-specific differences in the projective identification among the rs6090453 genotypes (*p* = 0.003); in the intermediate defense, reaction formation, and projective identification among the rs6011914 genotypes (*p* = 0.011, 0.010, and 0.011, respectively); and in the projective identification among the rs2427422 genotypes (*p* = 0.005) were found when the level of significance was adjusted by the Bonferroni correction. There was no significant difference in any of the defense scores among genotypes of any single nucleotide polymorphism in the total cohort or female subjects (all *p* > 0.017). The distributions of genotypes between the low and high score subgroups showed significant differences in the rs2427422 genotype distributions for help-rejecting complaining, regression, and projective identification (*p* = 0.010, 0.022, and 0.044, respectively). Significant differences were found between males and females in 10 defense mechanisms (all *P* < 0.05).

**Conclusions:** The gene variations in the *NTR1* polymorphisms were involved in the biological mechanisms of intermediate defense mechanisms, and this effect was influenced by sex.

## Introduction

The concept of the psychological defense mechanism is one of the cores in the psychoanalytic theory, which was first proposed by Freud (1). With the establishment and development of the personality structure model (id, ego, and superego) in the psychoanalytic school, the concept of the defense mechanism changed constantly (2). In the Diagnostic and Statistical Manual of Mental Disorders-Fourth Edition, defense mechanisms were defined as “automatic psychological processes that protect the individual against anxiety and from the awareness of internal or external dangers or stressors. Individuals are often unaware of these processes as they operate” (3). Psychological defense mechanisms represent a crucial component of individual capacity to maintain emotional homeostasis. Without them the conscious mind would be much more vulnerable to negatively charged emotional input, such as that pertaining to anxiety and sadness (4, 5).

In fact, until defense-related scales were developed, it had always been difficult to study defense mechanisms since they always worked at the unconscious level. Bond et al. developed and revised the self-reporting Defense Style Questionnaire 88 (DSQ-88) to assess possible conscious derivatives of defense mechanisms and to elicit manifestations of a subject's characteristic style of dealing with conflict, either consciously or unconsciously, based on the assumption that the subject was able to accurately comment on his/her behavior from a distance (6). The authors provided evidence for testing the reliability of the DSQ-88 by assessing psychiatric patients and healthy subjects (7–9). Since then, this questionnaire has been applied to a large body of clinical research (10–16).

The relationship of defense mechanisms with the psychopathology of mental disorders and abnormal behaviors is not been clearly understood, and in cases conflicting (17). Nevertheless, most studies have indicated strong evidence that adaptation of defense style correlated with mental health and that some diagnoses were correlated with specific defense patterns, for example, patients with depression or personality disorders tended to use more maladaptive defenses and less adaptive defenses while patients with anxiety disorders in general tended to use more immature and neurotic defenses (18, 19). Therefore, it is important and necessary to elucidate the determination of defenses.

There have been many previous studies on the social psychological factors of defense mechanisms in psychology; however, few have focused on biological factors. Moreover, the majority of previous studies focused on psychoanalytic and not statistical investigations (2). Using the data from twin-based studies, Andrews conducted a multivariate genetic analysis of three factors, including trait anxiety, locus of control and defense style, and demonstrated that defense style was substantially influenced by genetic factors and there was a significant loading (0.44) on a common genetic factor contributing to the variance of individual defenses (20). Therefore, biogenetic factors play a role in the determination of defense mechanisms. It is known that defense mechanisms are an important and enduring facet of personality (8) and that the dopamine system is closely related to personality traits (21–24). It was thus speculated that the dopamine system was associated with defenses. Two studies confirmed this hypothesis: Coming et al. demonstrated that the dopamine D2 receptor gene (*DRD2*) locus was the site of one of the factors that control defenses (25) and Huang et al. demonstrated that the *PPP1R1B* gene, encoding the dopamine- and cAMP-regulated phosphoprotein (DARPP-32), was one of the factors that was responsible for defenses (26).

In the central nervous system, neurotensin (NT) is a 13-amino acid multifunctional neurotransmitter and neuromodulator. Among the three NT receptor (NTR) subtypes, *NTR1* and *NTR2* are G protein-coupled receptors, and *NTR1* has a much higher affinity for NT than *NTR2* (27, 28). The *NTR1* gene is located on the 20q13 locus and consists of four exons and three introns (29). A large amount of anatomical, physiological, pharmacological and behavioral evidence has demonstrated that NT transmission modulates central dopaminergic functions (30–33). Therefore, the genes of the NT system may contribute to defense mechanisms by regulating dopamine neurotransmission. Moreover, our team has already demonstrated an association between *NTR1* gene single nucleotide polymorphisms (SNPs) and personality traits assessed by the Tridimensional Personality Questionnaire (TPQ) in Chinese Han subjects (22, 34). Therefore, we decided to test the hypothesis that the *NTR1* SNPs were associated with defenses.

**Figure 1:**
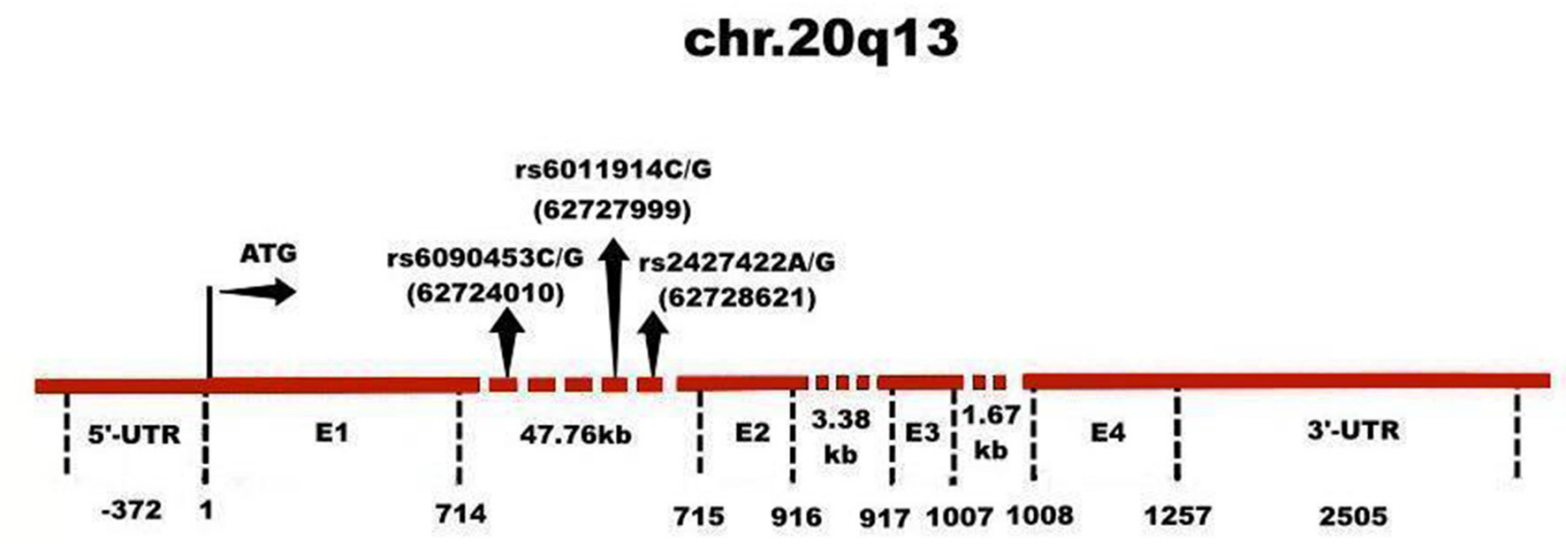
Genomic structure of NTR1 gene. The NTR1 gene has 4 exons (E) and 3 introns, whose sizes are indicated. The rs6090453C/G, rs6011914C/G, and rs2427422A/G polymorphisms of the NTR1 gene are located in intron 1, whose locations are indicated.

To our knowledge, none of the previous reports have found an association between *NTR1* gene polymorphisms and defense mechanisms measured by DSQ. In this study, we investigated whether genetic variants in the *NTR1* gene were associated with defenses in a large healthy Chinese Han population, and then analyzed any association by sex. The results would provide empirical evidence for the biological factors of defense mechanism. The three SNPs investigated (rs6090453, rs6011914, and rs2427422, Figure 1) were selected due to our previous research on personality traits (22, 34), copying styles (35), anxiety (36), schizophrenia (37), and alcohol dependence (38).

## Materials and Methods

### Subjects

All subjects were unrelated healthy Chinese-Han volunteers (*n* = 412, 196 males, 216 females) without psychiatric, neurological, or chronic physical illnesses. The age range was 19–58 years, while the mean ± standard deviation (SD) was 31.43 ± 8.16 years. There was no significant difference in age between the males and females (*p* = 0.187; Supplementary Table 1). The volunteers were recruited from healthy undergraduate and graduate students and staff of China Medical University, Shenyang, Liaoning Province, China, as well as healthy individuals undergoing physical examinations at the First Affiliated Hospital of China Medical University. Written informed consent was obtained from all participants. All the protocols in the present study were approved by the Ethics Committee of China Medical University (number of ethical approval: 2019-209-2).

Bond's DSQ-88 was translated and validated into the Chinese version in 1993, with no differences in meaning or content to that of the original version. The Chinese version of DSQ-88 evaluates 24 defense styles, which are classified into three factors of defense: immature or maladaptive (projection, passive aggression, acting out, help-rejecting complaining, fantasy, splitting, regression, and somatization), intermediate or neurotic (reaction formation, undoing, inhibition, withdrawal, idealization, pseudo-altruism, omnipotence, isolation, projective identification, denial, affiliation, consumption, and anticipation), and mature or adaptive (sublimation, suppression, and humor). All participants were asked to complete the questionnaire by themselves within 40 min and to check that all items had been scored. Following the completion of the questionnaire, 2 mL of venous blood from each participant was obtained for genotyping. Each defense style score was represented by the average of all the items representing the particular defense mechanism, while each defense factor score was derived from the mean of all the defense style scores belonging to the defense factor. According to the study of Bond et al. (6), a subject was considered to score high on a particular defense style and use it if his score was 0.5 SD above the mean on the particular defense. A cutting point of 0.5 SD provided the best discrimination between those who use a defense style and those who do not (6). Thus, subjects could be divided into low score (non-use) and high score (use) subgroups for each defense style based on whether the scores were above or below the cutting point.

### Polymorphism Genotyping

Amplification of gene fragments containing the SNPs rs6090453, rs6011914, and rs2427422 by polymerase chain reaction and subsequent identification of genotypes by restriction fragment length polymorphism analysis were carried out as described in our previous studies (22, 34–38).

### Statistical Analysis

Data are presented as the mean ± SD, frequency, or percentage. Data statistical analyses were conducted using SPSS^®^ version 17.0 software (SPSS Inc., Chicago, IL, USA). Potential genotypic associations of the three SNPs with DSQ-scores were detected by one-way analysis of variance (ANOVA) or by the non-parametric Kruskal-Wallis test, depending on homogeneity or heterogeneity of variance of data. Separate analysis by gender was also carried out. The Hardy-Weinberg equilibrium (HWE) and the differences in genotype frequencies between the low and high score subgroups were assessed by chi-square test. The differences in defense mechanisms between male and female subjects were compared by independent sample *t*-test. Linkage disequilibrium (LD) and haplotype analysis of the three *NTR1* gene polymorphisms were carried out using the free online software Haploview version 4.2 (http://www.broad.mit.edu/mpg/haploview). A *P* < 0.05 was regarded as statistically significant.

## Results

### HWE Results

The numbers of subjects with rs6090453 genotype CC, CG, and GG were 43, 177, and 192, respectively. The numbers of subjects with rs6011914 genotype GG, CG, and CC were 204, 176, and 32, respectively. The numbers of subjects with rs2427422 genotype GG, AG, and AA were 222, 168, and 22, respectively. The genotype distribution of the SNPs rs6090453 (χ^2^ = 0.054, *p* = 0.816), rs6011914 (χ^2^ = 0.496, *p* = 0.481), and rs2427422 (χ^2^ = 1.847, *p* = 0.174) did not deviate from the HWE in the experimental sample of 412 Chinese Han subjects (Supplementary Table 2). Moreover, for all three SNPs, the genotype frequencies were similar to those observed in other samples from the Han Chinese population (http://www.ncbi.nlm.nih.gov) (all *p* > 0.05). Therefore, this study sample was considered to be representative of the general Chinese Han population.

### LD and Haplotype Analysis Results

**Figure 2:**
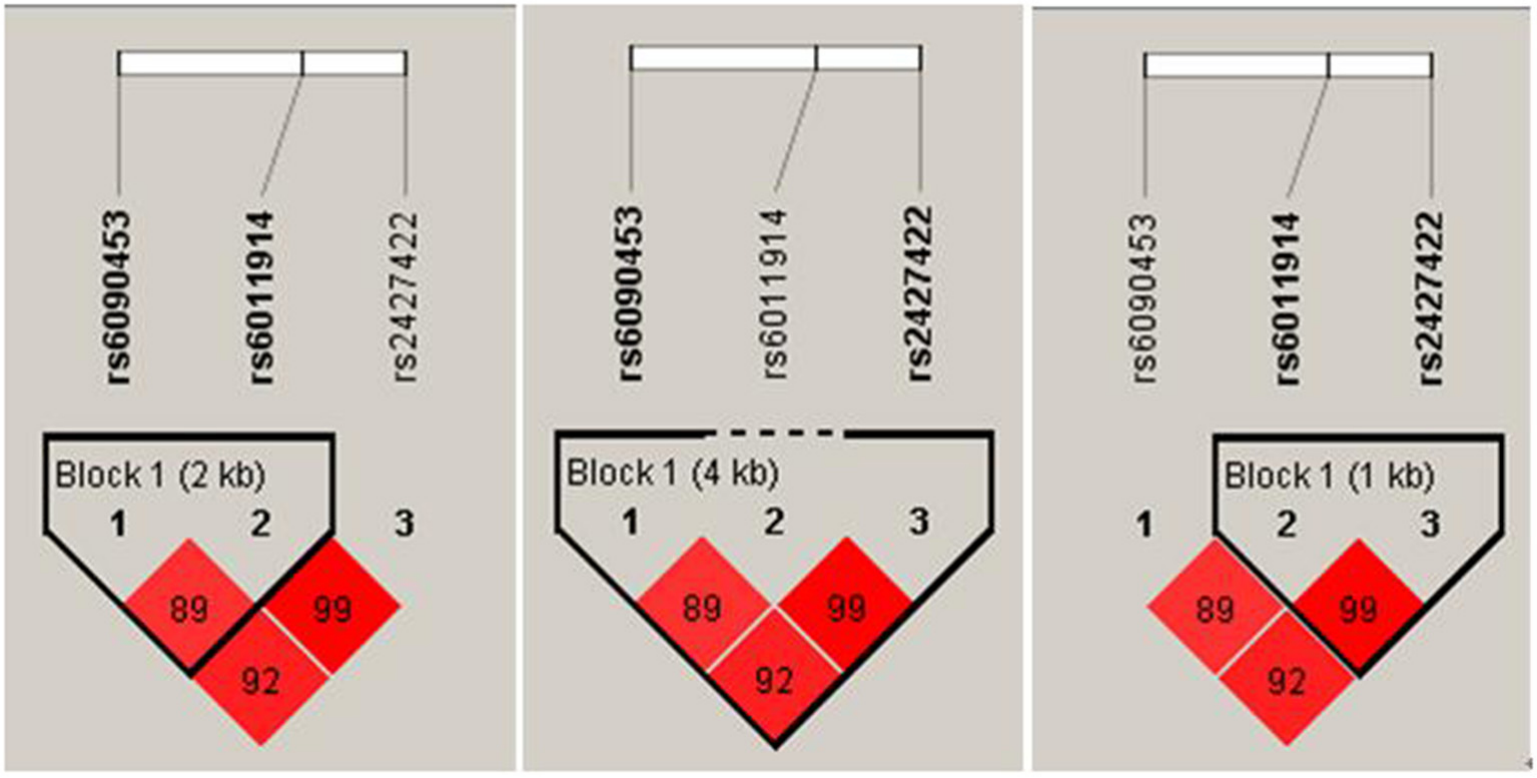
Linkage disequilibrium plots for the three analyzed NTR1 SNPs in healthy Chinese.

As shown in Figure 2, LDs between rs6090453C/G and rs6011914C/G (D′ = 0.89, *r*^2^ = 0.69), between rs6090453C/G and rs2427422A/G (D′ = 0.92, *r*^2^ = 0.63), and between rs6011914C/G and rs2427422A/G (D′ = 0.99, *r*^2^ = 0.83) were demonstrated.

The frequencies of haplotypes composed of the alleles in the three SNPs were shown in Table 1. The haplotype blocks with low frequencies (<1%) were rejected. The two most frequent haplotypes were GGG (0.659) and CCA (0.243). None of the frequencies of these five haplotypes differed significantly between the male and female samples (all *p* > 0.05).

**Table 1:** Distribution of haplotypes for the three analyzed *NTR1* SNPs between males and females.

**Haplotype**	**rs6090453**	**rs6011914**	**rs2427422**	**Frequency**	**Male, female ratio**	**χ^2^**	***p***
1	G	G	G	0.659	258.6:133.4, 284.6:147.4	0.001	0.979
2	C	C	A	0.243	97.6:294.4, 102.7:329.3	0.138	0.711
3	C	G	G	0.048	20.4:371.6, 19.4:412.6	0.228	0.633
4	C	C	G	0.027	10.0:382.0, 11.9:420.1	0.029	0.864
5	G	C	A	0.013	2.4:389.6, 8.3:423.7	2.700	0.100

### Effects of *NTR1* Gene Polymorphisms on DSQ Scores in the Total Cohort and in Male and Female Subjects

As shown in Table 2, all DSQ dimension scores among three genotypes of each SNP were compared. There was a marginal difference only in the score of help-rejecting complaining among the three genotypes of the rs2427422 polymorphism (*F* = 3.028, *p* = 0.050), but this difference was not significant when the level of significance was adjusted using Bonferroni correction [α′ = α/k, where α is the unadjusted significance level for pair-wise comparisons, k is the number of independent significance tests, and α′ (0.05/3 = 0.017) is the corrected significance level for multiple comparisons].

**Table 2:** Effects of NTR1 gene polymorphisms on DSQ scores.

**Defense mechanism**	**rs6090453**			**rs6011914**			**rs2427422**		
	**Genotype**	**Mean ± SD**	***P***	**Genotype**	**Mean ± SD**	***P***	**Genotype**	**Mean ± SD**	***P***
**Immature defense**	CC	3.59 ± 0.70	0.645	GG	3.68 ± 0.77	0.622	GG	3.68 ± 0.76	0.534
	CG	3.62 ± 0.76		CG	3.60 ± 0.72		AG	3.61 ± 0.74	
	GG	3.68 ± 0.75		CC	3.64 ± 0.76		AA	3.56 ± 0.70	
Projection	CC	2.74 ± 0.82	0.743	GG	2.82 ± 0.84	0.634	GG	2.84 ± 0.85	0.321
	CG	2.85 ± 0.84		CG	2.85 ± 0.82		AG	2.84 ± 0.82	
	GG	2.83 ± 0.82		CC	2.70 ± 0.82		AA	2.57 ± 0.66	
Passive aggression	CC	3.25 ± 1.14	0.860	GG	3.35 ± 1.03	0.442	GG	3.36 ± 1.01	0.525
	CG	3.30 ± 1.03		CG	3.24 ± 1.00		AG	3.24 ± 1.04	
	GG	3.34 ± 1.02		CC	3.45 ± 1.21		AA	3.38 ± 1.21	
Acting out	CC	4.04 ± 1.04	0.174	GG	4.35 ± 1.20	0.254	GG	4.32 ± 1.19	0.464
	CG	4.18 ± 1.26		CG	4.14 ± 1.28		AG	4.17 ± 1.30	
	GG	4.36 ± 1.24		CC	4.21 ± 1.14		AA	4.16 ± 1.18	
Help-rejecting complaining	CC	3.50 ± 1.08	0.615	GG	3.69 ± 1.26	0.188	GG	3.71 ± 1.24	0.050
	CG	3.70 ± 1.27		CG	3.72 ± 1.24		AG	3.70 ± 1.25	
	GG	3.68 ± 1.23		CC	3.29 ± 0.94		AA	3.04 ± 0.81	
Fantasy	CC	4.07 ± 2.06	0.192	GG	4.40 ± 2.10	0.141	GG	4.41 ± 2.11	0.114
	CG	4.03 ± 2.04		CG	3.98 ± 2.02		AG	3.98 ± 2.01	
	GG	4.41 ± 2.09		CC	4.28 ± 2.05		AA	4.00 ± 1.95	
Splitting	CC	4.60 ± 1.13	0.464	GG	4.39 ± 1.11	0.379	GG	4.39 ± 1.09	0.579
	CG	4.37 ± 1.20		CG	4.36 ± 1.16		AG	4.37 ± 1.22	
	GG	4.38 ± 1.06		CC	4.66 ± 1.10		AA	4.64 ± 0.80	
Regression	CC	4.12 ± 1.54	0.115	GG	4.15 ± 1.64	0.229	GG	4.13 ± 1.65	0.180
	CG	3.85 ± 1.52		CG	3.88 ± 1.51		AG	3.87 ± 1.48	
	GG	4.18 ± 1.64		CC	4.16 ± 1.52		AA	4.32 ± 1.63	
Somatization	CC	4.72 ± 1.56	0.957	GG	4.69 ± 1.51	0.847	GG	4.67 ± 1.50	0.980
	CG	4.65 ± 1.42		CG	4.61 ± 1.43		AG	4.65 ± 1.41	
	GG	4.66 ± 1.51		CC	4.73 ± 1.52		AA	4.61 ± 1.69	
**Intermediate defense**	CC	4.96 ± 0.93	0.527	GG	5.15 ± 0.94	0.517	GG	5.15 ± 0.93	0.656
	CG	5.12 ± 0.90		CG	5.09 ± 0.91		AG	5.07 ± 0.91	
	GG	5.14 ± 0.96		CC	4.96 ± 0.96		AA	5.05 ± 1.03	
Reaction formation	CC	5.40 ± 1.30	0.585	GG	5.63 ± 1.28	0.350	GG	5.64 ± 1.26	0.617
	CG	5.61 ± 1.22		CG	5.59 ± 1.25		AG	5.54 ± 1.28	
	GG	5.60 ± 1.31		CC	5.28 ± 1.23		AA	5.41 ± 1.28	
Undoing	CC	5.27 ± 1.48	0.400	GG	5.61 ± 1.32	0.376	GG	5.61 ± 1.35	0.412
	CG	5.52 ± 1.43		CG	5.47 ± 1.46		AG	5.42 ± 1.45	
	GG	5.59 ± 1.34		CC	5.28 ± 1.46		AA	5.48 ± 1.43	
Inhibition	CC	4.29 ± 1.17	0.921	GG	4.36 ± 1.18	0.896	GG	4.35 ± 1.18	0.989
	CG	4.34 ± 1.10		CG	4.32 ± 1.09		AG	4.34 ± 1.08	
	GG	4.37 ± 1.18		CC	4.41 ± 1.22		AA	4.38 ± 1.25	
Withdrawal	CC	4.32 ± 0.58	0.823	GG	4.36 ± 0.54	0.362	GG	4.36 ± 0.54	0.293
	CG	4.31 ± 0.51		CG	4.28 ± 0.53		AG	4.27 ± 0.53	
	GG	4.34 ± 0.56		CC	4.35 ± 0.59		AA	4.34 ± 0.61	
Idealization	CC	4.16 ± 1.13	0.734	GG	4.18 ± 0.96	0.065	GG	4.16 ± 0.98	0.296
	CG	4.06 ± 0.94		CG	3.98 ± 0.99		AG	4.01 ± 1.02	
	GG	4.13 ± 1.01		CC	4.32 ± 1.16		AA	4.24 ± 0.96	
Pseudo-altruism	CC	4.87 ± 1.12	0.803	GG	4.76 ± 1.23	0.961	GG	4.73 ± 1.23	0.908
	CG	4.74 ± 1.27		CG	4.73 ± 1.27		AG	4.78 ± 1.28	
	GG	4.74 ± 1.23		CC	4.78 ± 1.01		AA	4.74 ± 0.94	
Omnipotence	CC	4.85 ± 0.87	0.923	GG	4.91 ± 1.04	0.546	GG	4.91 ± 1.02	0.567
	CG	4.84 ± 0.98		CG	4.80 ± 0.95		AG	4.80 ± 0.96	
	GG	4.88 ± 1.04		CC	4.90 ± 0.95		AA	4.85 ± 0.98	
Isolation	CC	5.02 ± 1.49	0.329	GG	4.91 ± 1.47	0.225	GG	4.91 ± 1.44	0.144
	CG	4.71 ± 1.45		CG	4.69 ± 1.44		AG	4.67 ± 1.47	
	GG	4.88 ± 1.44		CC	5.05 ± 1.40		AA	5.15 ± 1.36	
Projective identification	CC	4.86 ± 1.88	0.869	GG	4.91 ± 1.63	0.423	GG	4.93 ± 1.64	0.108
	CG	4.75 ± 1.63		CG	4.68 ± 1.68		AG	4.60 ± 1.67	
	GG	4.83 ± 1.66		CC	4.78 ± 1.83		AA	5.07 ± 1.84	
Denial	CC	5.09 ± 1.97	0.478	GG	4.81 ± 1.96	0.595	GG	4.79 ± 1.96	0.431
	CG	4.99 ± 1.91		CG	5.01 ± 1.94		AG	5.04 ± 1.92	
	GG	4.79 ± 1.99		CC	5.00 ± 1.97		AA	5.09 ± 2.11	
Affiliation	CC	3.50 ± 0.73	0.689	GG	3.65 ± 0.96	0.645	GG	3.67 ± 0.94	0.497
	CG	3.63 ± 0.87		CG	3.60 ± 0.86		AG	3.56 ± 0.87	
	GG	3.63 ± 0.96		CC	3.51 ± 0.76		AA	3.53 ± 0.69	
Consumption	CC	3.82 ± 0.85	0.391	GG	3.90 ± 0.91	0.560	GG	3.92 ± 0.92	0.128
	CG	3.80 ± 0.83		CG	3.84 ± 0.86		AG	3.82 ± 0.86	
	GG	3.92 ± 0.95		CC	3.73 ± 0.92		AA	3.55 ± 0.68	
Anticipation	CC	2.42 ± 1.69	0.640	GG	2.27 ± 1.53	0.738	GG	2.28 ± 1.54	0.650
	CG	2.19 ± 1.38		CG	2.15 ± 1.38		AG	2.15 ± 1.37	
	GG	2.20 ± 1.50		CC	2.25 ± 1.61		AA	2.09 ± 1.51	
**Mature defense**	CC	4.40 ± 1.07	0.227	GG	4.68 ± 0.93	0.813	GG	4.67 ± 0.95	0.809
	CG	4.68 ± 0.98		CG	4.62 ± 1.01		AG	4.64 ± 1.02	
	GG	4.68 ± 0.97		CC	4.63 ± 1.16		AA	4.53 ± 1.16	
Sublimation	CC	4.40 ± 1.55	0.547	GG	4.52 ± 1.49	0.362	GG	4.54 ± 1.49	0.486
	CG	4.68 ± 1.52		CG	4.74 ± 1.56		AG	4.73 ± 1.55	
	GG	4.61 ± 1.54		CC	4.55 ± 1.61		AA	4.64 ± 1.77	
Suppression	CC	3.58 ± 1.11	0.720	GG	3.62 ± 1.11	0.799	GG	3.63 ± 1.19	0.680
	CG	3.53 ± 1.14		CG	3.54 ± 1.20		AG	3.53 ± 1.10	
	GG	3.63 ± 1.17		CC	3.57 ± 1.09		AA	3.59 ± 1.12	
Humor	CC	5.76 ± 1.29	0.597	GG	5.66 ± 1.44	0.667	GG	5.70 ± 1.42	0.715
	CG	5.77 ± 1.38		CG	5.72 ± 1.32		AG	5.68 ± 1.32	
	GG	5.63 ± 1.40		CC	5.89 ± 1.34		AA	5.93 ± 1.42	

*P-values without underline were the results of ANOVA*.

The differences in all DSQ dimension scores among the genotypes of the three polymorphisms were then analyzed separately by sex. We demonstrated significant male-specific differences in the intermediate defense, reaction formation, projective identification, affiliation, mature defense, sublimation, and suppression scores among the rs6090453 genotypes (*F* = 3.445, 3.131, 5.982, 4.023, 3.552, 3.892, and 3.061, respectively; *p* = 0.034, 0.046, 0.003, 0.019, 0.031, 0.022, and 0.049, respectively); in the intermediate defense, reaction formation, undoing, and projective identification scores among the rs6011914 genotypes (*F* = 4.651, 4.760, 3.999, and 4.631, respectively; *p* = 0.011, 0.010, 0.020, and 0.011, respectively); and in the projective identification score among the rs2427422 genotypes (*F* = 5.433, *p* = 0.005; Table 3). Moreover, the male-specific differences in the projective identification score among the rs6090453 genotypes, in the intermediate defense, reaction formation, and projective identification scores among the rs6011914 genotypes, and in the projective identification score among the rs2427422 genotypes were still significant (all *P* < 0.017) when the level of significance was adjusted by the Bonferroni correction.

**Table 3:** Effects of NTR1 gene polymorphisms on DSQ scores in males.

**Defense mechanism**	**rs6090453**			**rs6011914**			**rs2427422**		
	**Genotype**	**Mean ± SD**	***P***	**Genotype**	**Mean ± SD**	***P***	**Genotype**	**Mean ± SD**	***P***
**Immature defense**	CC	3.48 ± 0.70	0.577	GG	3.65 ± 0.77	0.708	GG	3.67 ± 0.77	0.655
	CG	3.67 ± 0.73		CG	3.66 ± 0.70		AG	3.63 ± 0.70	
	GG	3.66 ± 0.75		CC	3.48 ± 0.76		AA	3.43 ± 0.78	
Projection	CC	2.68 ± 0.78	0.438	GG	2.91 ± 0.84	0.530	GG	2.95 ± 0.86	0.360
	CG	2.94 ± 0.77		CG	2.93 ± 0.77		AG	2.88 ± 0.73	
	GG	2.91 ± 0.85		CC	2.66 ± 0.78		AA	2.54 ± 0.83	
Passive aggression	CC	3.08 ± 1.23	0.266	GG	3.29 ± 0.86	0.459	GG	3.33 ± 0.85	0.729
	CG	3.45 ± 1.02		CG	3.43 ± 1.03		AG	3.38 ± 1.05	
	GG	3.29 ± 0.85		CC	3.14 ± 1.38		AA	3.10 ± 1.59	
Acting out	CC	3.87 ± 0.95	0.425	GG	4.25 ± 1.13	0.842	GG	4.23 ± 1.12	0.949
	CG	4.22 ± 1.20		CG	4.17 ± 1.22		AG	4.18 ± 1.21	
	GG	4.25 ± 1.15		CC	4.08 ± 0.92		AA	4.15 ± 1.12	
Help-rejecting complaining	CC	3.51 ± 1.01	0.297	GG	3.56 ± 1.11	0.060	GG	3.59 ± 1.09	0.144
	CG	3.78 ± 1.28		CG	3.83 ± 1.28		AG	3.77 ± 1.29	
	GG	3.52 ± 1.09		CC	3.08 ± 0.70		AA	2.96 ± 0.68	
Fantasy	CC	4.00 ± 2.19	0.883	GG	4.04 ± 1.94	0.836	GG	4.10 ± 1.97	0.645
	CG	3.91 ± 1.95		CG	3.90 ± 1.90		AG	3.84 ± 1.90	
	GG	4.06 ± 1.91		CC	4.15 ± 2.41		AA	4.13 ± 2.36	
Splitting	CC	4.69 ± 1.34	0.552	GG	4.42 ± 1.13	0.640	GG	4.45 ± 1.11	0.985
	CG	4.37 ± 1.12		CG	4.41 ± 1.09		AG	4.42 ± 1.18	
	GG	4.45 ± 1.08		CC	4.72 ± 1.33		AA	4.46 ± 0.62	
Regression	CC	3.71 ± 1.67	0.657	GG	3.91 ± 1.54	0.815	GG	3.92 ± 1.56	0.769
	CG	3.78 ± 1.35		CG	3.82 ± 1.37		AG	3.76 ± 1.31	
	GG	3.96 ± 1.56		CC	3.65 ± 1.69		AA	3.94 ± 2.01	
Somatization	CC	4.58 ± 1.57	0.571	GG	4.81 ± 1.59	0.617	GG	4.78 ± 1.60	0.837
	CG	4.63 ± 1.32		CG	4.60 ± 1.39		AG	4.65 ± 1.34	
	GG	4.84 ± 1.63		CC	4.81 ± 1.27		AA	4.69 ± 1.53	
**Intermediate defense**	CC	4.52 ± 0.75	0.034	GG	4.97 ± 0.93	0.011	GG	5.01 ± 0.91	0.162
	CG	5.13 ± 0.92		CG	5.17 ± 0.91		AG	5.08 ± 0.95	
	GG	5.00 ± 0.95		CC	4.36 ± 0.77		AA	4.43 ± 0.90	
Reaction formation	CC	4.93 ± 1.02	0.046	GG	5.60 ± 1.29	0.010	GG	5.65 ± 1.26	0.267
	CG	5.74 ± 1.28		CG	5.73 ± 1.29		AG	5.58 ± 1.35	
	GG	5.57 ± 1.33		CC	4.56 ± 0.95		AA	4.88 ± 1.01	
Undoing	CC	4.87 ± 1.22	0.083	GG	5.39 ± 1.28	0.020	GG	5.45 ± 1.30	0.246
	CG	5.59 ± 1.30		CG	5.67 ± 1.27		AG	5.55 ± 1.27	
	GG	5.46 ± 1.27		CC	4.65 ± 1.20		AA	4.75 ± 1.31	
Inhibition	CC	3.86 ± 1.05	0.456	GG	4.06 ± 1.15	0.410	GG	4.07 ± 1.11	0.313
	CG	4.21 ± 1.10		CG	4.26 ± 1.09		AG	4.26 ± 1.13	
	GG	4.12 ± 1.17		CC	3.95 ± 1.25		AA	3.75 ± 1.31	
Withdrawal	CC	4.02 ± 0.49	0.093	GG	4.27 ± 0.52	0.540	GG	4.31 ± 0.53	0.263
	CG	4.30 ± 0.52		CG	4.28 ± 0.52		AG	4.24 ± 0.51	
	GG	4.29 ± 0.52		CC	4.11 ± 0.53		AA	4.01 ± 0.54	
Idealization	CC	4.00 ± 1.23	0.911	GG	4.09 ± 0.93	0.501	GG	4.11 ± 0.94	0.725
	CG	4.04 ± 0.96		CG	3.98 ± 0.96		AG	4.00 ± 1.04	
	GG	4.09 ± 0.92		CC	4.28 ± 1.33		AA	3.98 ± 0.57	
Pseudo-altruism	CC	4.68 ± 0.83	0.892	GG	4.81 ± 1.10	0.831	GG	4.80 ± 1.11	0.598
	CG	4.82 ± 1.12		CG	4.81 ± 1.14		AG	4.83 ± 1.10	
	GG	4.79 ± 1.12		CC	4.62 ± 0.72		AA	4.42 ± 0.73	
Omnipotence	CC	4.55 ± 0.84	0.828	GG	4.62 ± 0.97	0.913	GG	4.65 ± 0.97	0.952
	CG	4.67 ± 0.92		CG	4.67 ± 0.86		AG	4.62 ± 0.83	
	GG	4.62 ± 0.93		CC	4.57 ± 0.89		AA	4.58 ± 1.04	
Isolation	CC	4.51 ± 1.42	0.966	GG	4.53 ± 1.35	0.737	GG	4.59 ± 1.33	0.664
	CG	4.54 ± 1.42		CG	4.54 ± 1.41		AG	4.49 ± 1.44	
	GG	4.59 ± 1.34		CC	4.85 ± 1.41		AA	4.92 ± 1.44	
Projective identification	CC	3.55 ± 1.31	0.003	GG	4.82 ± 1.52	0.011	GG	4.86 ± 1.51	0.005
	CG	4.51 ± 1.46		CG	4.40 ± 1.45		AG	4.27 ± 1.43	
	GG	4.83 ± 1.51		CC	3.62 ± 1.33		AA	3.63 ± 1.41	
Denial	CC	4.53 ± 2.01	0.668	GG	4.78 ± 1.89	0.538	GG	4.80 ± 1.87	0.786
	CG	4.96 ± 1.93		CG	5.01 ± 1.96		AG	4.96 ± 1.97	
	GG	4.84 ± 1.91		CC	4.46 ± 1.94		AA	4.63 ± 2.20	
Affiliation	CC	3.37 ± 0.61	0.019	GG	3.59 ± 0.92	0.065	GG	3.65 ± 0.93	0.477
	CG	3.86 ± 0.79		CG	3.84 ± 0.81		AG	3.76 ± 0.81	
	GG	3.57 ± 0.95		CC	3.37 ± 0.53		AA	3.44 ± 0.48	
Consumption	CC	3.83 ± 1.04	0.888	GG	3.84 ± 0.75	0.831	GG	3.89 ± 0.81	0.301
	CG	3.81 ± 0.79		CG	3.86 ± 0.80		AG	3.82 ± 0.79	
	GG	3.87 ± 0.76		CC	3.71 ± 1.19		AA	3.44 ± 0.78	
Anticipation	CC	2.37 ± 1.83	0.558	GG	2.21 ± 1.44	0.374	GG	2.26 ± 1.48	0.417
	CG	2.36 ± 1.30		CG	2.40 ± 1.42		AG	2.33 ± 1.38	
	GG	2.14 ± 1.48		CC	1.85 ± 1.52		AA	1.63 ± 1.41	
**Mature defense**	CC	4.04 ± 1.14	0.031	GG	4.68 ± 0.87	0.757	GG	4.70 ± 0.88	0.084
	CG	4.63 ± 1.05		CG	4.55 ± 1.10		AG	4.55 ± 1.10	
	GG	4.69 ± 0.87		CC	4.36 ± 1.15		AA	3.92 ± 1.06	
Sublimation	CC	3.58 ± 1.25	0.022	GG	4.38 ± 1.38	0.162	GG	4.37 ± 1.43	0.360
	CG	4.62 ± 1.56		CG	4.63 ± 1.63		AG	4.60 ± 1.57	
	GG	4.48 ± 1.43		CC	3.85 ± 1.36		AA	3.94 ± 1.64	
Suppression	CC	3.44 ± 0.97	0.049	GG	3.96 ± 1.20	0.220	GG	3.96 ± 1.21	0.172
	CG	3.69 ± 1.12		CG	3.68 ± 1.10		AG	3.65 ± 1.07	
	GG	4.03 ± 1.19		CC	3.64 ± 1.03		AA	3.63 ± 1.04	
Humor	CC	5.58 ± 1.52	0.705	GG	5.48 ± 1.52	0.783	GG	5.55 ± 1.52	0.982
	CG	5.63 ± 1.46		CG	5.59 ± 1.39		AG	5.53 ± 1.38	
	GG	5.45 ± 1.48		CC	5.73 ± 1.72		AA	5.63 ± 1.90	

*P-values with underline were the results of the non-parametric Kruskal–Wallis test, while P-values without underline were the results of ANOVA*.

At the same time, we demonstrated significant female-specific difference in the projective identification score among between the rs6090453 genotypes (*F* = 3.580, *p* = 0.030), and in the passive aggression, intermediated defense, and affiliation scores among the rs6011914 genotypes (*F* = 3.775, 3.080, and 3.273, respectively; *p* = 0.024, 0.048, and 0.040, respectively; Table 4). However, all these female-specific differences in defense scores among the genotypes of the three SNPs were not significant when the level of significance was adjusted by the Bonferroni correction.

**Table 4:** Effects of NTR1 gene polymorphisms on DSQ scores in females.

**Defense mechanism**	**rs6090453**			**rs6011914**			**rs2427422**		
	**Genotype**	**Mean ± SD**	***P***	**Genotype**	**Mean ± SD**	***P***	**Genotype**	**Mean ± SD**	***P***
**Immature defense**	CC	3.68 ± 0.70	0.461	GG	3.70 ± 0.78	0.281	GG	3.69 ± 0.76	0.588
	CG	3.56 ± 0.79		CG	3.54 ± 0.73		AG	3.58 ± 0.78	
	GG	3.70 ± 0.75		CC	3.75 ± 0.76		AA	3.63 ± 0.66	
Projection	CC	2.78 ± 0.86	0.982	GG	2.74 ± 0.84	0.945	GG	2.75 ± 0.83	0.660
	CG	2.75 ± 0.90		CG	2.78 ± 0.86		AG	2.80 ± 0.91	
	GG	2.76 ± 0.80		CC	2.73 ± 0.86		AA	2.58 ± 0.58	
Passive aggression	CC	3.38 ± 1.06	0.283	GG	3.40 ± 1.17	0.024	GG	3.38 ± 1.13	0.129
	CG	3.14 ± 1.01		CG	3.06 ± 0.95		AG	3.10 ± 1.03	
	GG	3.38 ± 1.15		CC	3.66 ± 1.06		AA	3.54 ± 0.97	
Acting out	CC	4.17 ± 1.10	0.202	GG	4.44 ± 1.27	0.201	GG	4.40 ± 1.25	0.414
	CG	4.13 ± 1.33		CG	4.11 ± 1.34		AG	4.16 ± 1.38	
	GG	4.45 ± 1.31		CC	4.31 ± 1.28		AA	4.17 ± 1.25	
Help-rejecting complaining	CC	3.49 ± 1.16	0.395	GG	3.80 ± 1.38	0.391	GG	3.81 ± 1.35	0.114
	CG	3.61 ± 1.25		CG	3.62 ± 1.20		AG	3.62 ± 1.21	
	GG	3.81 ± 1.33		CC	3.44 ± 1.07		AA	3.09 ± 0.90	
Fantasy	CC	4.13 ± 2.01	0.180	GG	4.73 ± 2.20	0.098	GG	4.68 ± 2.20	0.139
	CG	4.16 ± 2.13		CG	4.07 ± 2.13		AG	4.13 ± 2.12	
	GG	4.70 ± 2.19		CC	4.37 ± 1.83		AA	3.93 ± 1.77	
Splitting	CC	4.53 ± 0.96	0.707	GG	4.36 ± 1.09	0.559	GG	4.34 ± 1.07	0.433
	CG	4.37 ± 1.29		CG	4.30 ± 1.23		AG	4.32 ± 1.27	
	GG	4.31 ± 1.05		CC	4.61 ± 0.95		AA	4.74 ± 0.89	
Regression	CC	4.44 ± 1.38	0.133	GG	4.36 ± 1.71	0.136	GG	4.32 ± 1.70	0.256
	CG	3.92 ± 1.68		CG	3.93 ± 1.65		AG	3.97 ± 1.63	
	GG	4.37 ± 1.69		CC	4.50 ± 1.33		AA	4.54 ± 1.41	
Somatization	CC	4.83 ± 1.59	0.553	GG	4.58 ± 1.43	0.948	GG	4.58 ± 1.42	0.931
	CG	4.67 ± 1.53		CG	4.63 ± 1.47		AG	4.66 ± 1.49	
	GG	4.51 ± 1.39		CC	4.68 ± 1.70		AA	4.57 ± 1.83	
**Intermediate defense**	CC	5.31 ± 0.93	0.440	GG	5.32 ± 0.91	0.048	GG	5.27 ± 0.94	0.171
	CG	5.10 ± 0.88		CG	5.01 ± 0.92		AG	5.05 ± 0.88	
	GG	5.25 ± 0.95		CC	5.37 ± 0.87		AA	5.41 ± 0.94	
Reaction formation	CC	5.76 ± 1.40	0.545	GG	5.66 ± 1.29	0.397	GG	5.62 ± 1.27	0.727
	CG	5.48 ± 1.14		CG	5.45 ± 1.21		AG	5.50 ± 1.20	
	GG	5.63 ± 1.29		CC	5.77 ± 1.17		AA	5.72 ± 1.35	
Undoing	CC	5.58 ± 1.61	0.519	GG	5.81 ± 1.34	0.081	GG	5.74 ± 1.38	0.127
	CG	5.44 ± 1.56		CG	5.28 ± 1.60		AG	5.29 ± 1.61	
	GG	5.69 ± 1.39		CC	5.71 ± 1.49		AA	5.89 ± 1.36	
Inhibition	CC	4.64 ± 1.16	0.735	GG	4.63 ± 1.15	0.217	GG	4.59 ± 1.20	0.461
	CG	4.46 ± 1.08		CG	4.38 ± 1.09		AG	4.42 ± 1.03	
	GG	4.57 ± 1.16		CC	4.72 ± 1.12		AA	4.74 ± 1.10	
Withdrawal	CC	4.56 ± 0.53	0.157	GG	4.43 ± 0.55	0.080	GG	4.41 ± 0.56	0.244
	CG	4.31 ± 0.51		CG	4.28 ± 0.55		AG	4.30 ± 0.55	
	GG	4.39 ± 0.60		CC	4.52 ± 0.59		AA	4.53 ± 0.59	
Idealization	CC	4.29 ± 1.05	0.664	GG	4.25 ± 0.98	0.109	GG	4.20 ± 1.02	0.329
	CG	4.09 ± 0.93		CG	3.98 ± 1.03		AG	4.03 ± 1.00	
	GG	4.16 ± 1.08		CC	4.35 ± 1.07		AA	4.39 ± 1.12	
Pseudo-altruism	CC	5.01 ± 1.30	0.497	GG	4.72 ± 1.35	0.785	GG	4.67 ± 1.32	0.774
	CG	4.65 ± 1.41		CG	4.66 ± 1.39		AG	4.73 ± 1.44	
	GG	4.69 ± 1.31		CC	4.89 ± 1.18		AA	4.93 ± 1.02	
Omnipotence	CC	5.08 ± 0.84	0.860	GG	5.17 ± 1.04	0.242	GG	5.12 ± 1.02	0.603
	CG	5.01 ± 1.01		CG	4.92 ± 1.02		AG	4.98 ± 1.06	
	GG	5.09 ± 1.08		CC	5.13 ± 0.94		AA	5.01 ± 0.94	
Isolation	CC	5.43 ± 1.45	0.237	GG	5.24 ± 1.49	0.132	GG	5.19 ± 1.48	0.239
	CG	4.89 ± 1.48		CG	4.83 ± 1.46		AG	4.85 ± 1.48	
	GG	5.13 ± 1.48		CC	5.19 ± 1.41		AA	5.29 ± 1.35	
Projective identification	CC	5.90 ± 1.61	0.030	GG	4.99 ± 1.73	0.358	GG	4.99 ± 1.75	0.162
	CG	5.01 ± 1.77		CG	4.95 ± 1.83		AG	4.93 ± 1.83	
	GG	4.83 ± 1.78		CC	5.58 ± 1.72		AA	5.89 ± 1.55	
Denial	CC	5.54 ± 1.86	0.180	GG	4.83 ± 2.03	0.527	GG	4.79 ± 2.04	0.388
	CG	5.03 ± 1.89		CG	5.00 ± 1.93		AG	5.11 ± 1.87	
	GG	4.75 ± 2.06		CC	5.37 ± 1.95		AA	5.36 ± 2.10	
Affiliation	CC	3.61 ± 0.81	0.098	GG	3.70 ± 0.99	0.040	GG	3.68 ± 0.96	0.059
	CG	3.39 ± 0.90		CG	3.37 ± 0.85		AG	3.37 ± 0.90	
	GG	3.68 ± 0.97		CC	3.60 ± 0.89		AA	3.58 ± 0.79	
Consumption	CC	3.81 ± 0.69	0.597	GG	3.95 ± 1.03	0.517	GG	3.95 ± 1.01	0.360
	CG	3.78 ± 0.87		CG	3.82 ± 0.92		AG	3.82 ± 0.93	
	GG	3.96 ± 1.07		CC	3.74 ± 0.71		AA	3.61 ± 0.65	
Anticipation	CC	2.46 ± 1.61	0.348	GG	2.33 ± 1.61	0.094	GG	2.29 ± 1.59	0.298
	CG	2.01 ± 1.43		CG	1.92 ± 1.29		AG	1.98 ± 1.33	
	GG	2.25 ± 1.52		CC	2.53 ± 1.65		AA	2.36 ± 1.55	
**Mature defense**	CC	4.69 ± 0.94	0.900	GG	4.68 ± 0.99	0.869	GG	4.64 ± 1.00	0.627
	CG	4.73 ± 0.90		CG	4.68 ± 0.92		AG	4.73 ± 0.92	
	GG	4.66 ± 1.05		CC	4.81 ± 1.17		AA	4.88 ± 1.09	
Sublimation	CC	5.04 ± 1.47	0.658	GG	4.66 ± 1.58	0.518	GG	4.68 ± 1.54	0.588
	CG	4.75 ± 1.48		CG	4.85 ± 1.49		AG	4.86 ± 1.52	
	GG	4.73 ± 1.62		CC	5.03 ± 1.62		AA	5.04 ± 1.77	
Suppression	CC	3.70 ± 1.22	0.308	GG	3.32 ± 0.93	0.848	GG	3.34 ± 1.09	0.751
	CG	3.37 ± 1.15		CG	3.42 ± 1.29		AG	3.39 ± 1.13	
	GG	3.31 ± 1.05		CC	3.53 ± 1.16		AA	3.57 ± 1.20	
Humor	CC	5.90 ± 1.08	0.739	GG	5.82 ± 1.34	0.854	GG	5.82 ± 1.32	0.728
	CG	5.91 ± 1.28		CG	5.83 ± 1.26		AG	5.83 ± 1.26	
	GG	5.77 ± 1.33		CC	6.00 ± 1.04		AA	6.11 ± 1.10	

*P-values with underline were the results of the non-parametric Kruskal–Wallis test, while P-values without underline were the results of ANOVA*.

### Comparison of the Genotype Distributions Between the High and Low Score Subgroups

As shown in Table 5, the genotypes of the three SNPs between the high and low score subgroups for three defense factors and 24 defense styles were compared. We found significant differences in the rs2427422 genotype distributions between two subgroups for help-rejecting complaining, regression, and projective identification (χ^2^ = 9.182, 7.665, and 6.236, *p* = 0.010, 0.022, and 0.044, respectively).

**Table 5:** Comparison of genotype distributions of NTR1 gene polymorphisms between high- and low-score subgroups.

**Defense mechanism**	**Cutting point**	**rs6090453**				**rs6011914**				**rs2427422**			
		**Genotype**	**High**	**Low**	***P***	**Genotype**	**High**	**Low**	***P***	**Genotype**	**High**	**Low**	***P***
			***N*** **%**	***N*** **%**			***N*** **%**	***N*** **%**			***N*** **%**	***N*** **%**	
**Immature defense**	4.02	CC	9 8.0	34 11.3	0.580	GG	56 50.0	148 49.3	0.958	GG	62 55.4	160 53.3	0.617
		CG	51 45.5	126 42.0		CG	48 42.9	128 42.7		AG	46 41.1	122 40.7	
		GG	52 46.4	140 46.7		CC	8 7.1	24 8.0		AA	4 3.6	18 6.0	
Projection	3.24	CC	13 11.3	30 10.1	0.909	GG	54 47.0	150 50.5	0.482	GG	63 54.8	159 53.5	0.578
		CG	50 43.5	127 42.8		CG	54 47.0	122 41.1		AG	48 41.7	120 40.4	
		GG	52 45.2	140 47.1		CC	7 6.1	25 8.4		AA	4 3.5	18 6.1	
Passive aggression	3.83	CC	8 7.9	35 11.3	0.633	GG	53 52.5	151 48.6	0.762	GG	58 57.4	164 52.7	0.624
		CG	45 44.6	132 42.4		CG	40 39.6	136 43.7		AG	39 38.6	129 41.5	
		GG	48 47.5	144 46.3		CC	8 7.9	24 7.7		AA	4 4.0	18 5.8	
Acting out	4.87	CC	10 8.2	33 11.4	0.518	GG	64 52.5	140 48.3	0.740	GG	68 55.7	154 53.1	0.828
		CG	51 41.8	126 43.4		CG	49 40.2	127 43.8		AG	47 38.5	121 41.7	
		GG	61 50.0	131 45.2		CC	9 7.4	23 7.9		AA	7 5.7	15 5.2	
Help-rejecting complaining	4.28	CC	9 8.3	34 11.2	0.402	GG	56 51.9	148 48.7	0.187	GG	69 63.9	153 50.3	0.010
		CG	43 39.8	134 44.1		CG	43 39.8	133 43.8		AG	38 35.2	130 42.8	
		GG	56 51.9	136 44.7		CC	4 3.7	28 9.2		AA	1 0.9	21 6.9	
Fantasy	5.25	CC	13 10.9	30 10.2	0.192	GG	63 52.9	141 48.1	0.405	GG	70 58.8	152 51.9	0.434
		CG	43 36.1	134 45.7		CG	45 37.8	131 44.7		AG	43 36.1	125 42.7	
		GG	63 52.9	129 44.0		CC	11 9.2	21 7.2		AA	6 5.0	16 5.5	
Splitting	4.96	CC	14 10.6	29 10.4	0.935	GG	72 54.5	132 47.1	0.356	GG	77 58.3	145 51.8	0.440
		CG	55 41.7	122 43.6		CG	50 37.9	126 45.0		AG	48 36.4	120 42.9	
		GG	63 47.7	129 46.1		CC	10 7.6	22 7.9		AA	7 5.3	15 5.4	
Regression	4.82	CC	11 8.5	32 11.3	0.068	GG	75 58.1	129 45.6	0.061	GG	82 63.6	140 49.5	0.022
		CG	47 36.4	130 45.9		CG	46 35.7	130 45.9		AG	40 31.0	128 45.2	
		GG	71 55.0	121 42.8		CC	8 62.0	24 84.8		AA	7 5.4	15 5.3	
Somatization	5.40	CC	15 11.2	28 10.1	0.845	GG	69 51.5	135 48.6	0.401	GG	76 56.7	146 52.5	0.598
		CG	55 41.0	122 43.9		CG	52 38.8	124 44.6		AG	50 37.3	118 42.4	
		GG	64 47.8	128 46.0		CC	13 9.7	19 6.8		AA	8 6.0	14 5.0	
**Intermediate defense**	5.57	CC	13 9.5	30 10.9	0.674	GG	70 51.1	134 48.7	0.895	GG	77 56.2	145 52.7	0.491
		CG	56 40.9	121 44.0		CG	57 41.6	119 43.3		AG	51 37.2	117 42.5	
		GG	68 49.6	124 45.1		CC	10 7.3	22 8.0		AA	9 6.6	13 4.7	
Reaction formation	6.22	CC	13 9.4	30 10.9	0.812	GG	69 50.0	135 49.3	0.562	GG	75 54.3	147 53.6	0.817
		CG	58 42.0	119 43.4		CG	61 44.2	115 42.0		AG	57 41.3	111 40.5	
		GG	67 48.6	125 45.6		CC	8 5.8	24 8.8		AA	6 4.3	16 5.8	
Undoing	6.22	CC	14 10.8	29 10.3	0.708	GG	65 50.0	139 49.3	0.991	GG	72 55.4	150 53.2	0.754
		CG	52 40.0	125 44.3		CG	55 42.3	121 42.9		AG	50 38.5	118 41.8	
		GG	64 49.2	128 45.4		CC	10 7.7	22 7.8		AA	8 6.2	14 49.6	
Inhibition	4.92	CC	15 10.8	28 10.3	0.735	GG	71 51.1	133 48.7	0.530	GG	75 54.0	147 53.8	0.748
		CG	56 40.3	121 44.3		CG	55 39.6	121 44.3		AG	55 39.6	113 41.4	
		GG	68 48.9	124 45.4		CC	13 9.4	19 7.0		AA	9 6.5	13 47.6	
Withdrawal	4.59	CC	15 11.9	28 9.8	0.620	GG	66 52.4	138 48.3	0.565	GG	72 57.1	150 52.4	0.634
		CG	55 41.7	122 43.6		CG	50 37.9	126 45.0		AG	48 36.4	120 42.9	
		GG	63 47.7	129 46.1		CC	10 7.6	22 7.9		AA	7 5.3	15 5.4	
Idealization	4.60	CC	14 9.9	29 10.7	0.604	GG	78 54.9	126 46.7	0.192	GG	83 58.5	139 51.5	0.345
		CG	57 40.1	120 44.4		CG	52 36.6	124 45.9		AG	51 35.9	117 43.3	
		GG	71 50.0	121 44.8		CC	12 8.5	20 7.4		AA	8 5.6	14 5.2	
Pseudo-altruism	5.37	CC	12 11.9	31 10.0	0.377	GG	48 47.5	156 50.2	0.897	GG	51 50.5	171 55.0	0.673
		CG	48 47.5	129 41.5		CG	45 44.6	131 42.1		AG	45 44.6	123 39.5	
		GG	41 40.6	151 48.6		CC	8 7.9	24 7.7		AA	5 5.0	17 5.5	
Omnipotence	5.36	CC	11 8.3	32 11.5	0.607	GG	71 53.4	133 47.7	0.546	GG	75 56.4	147 52.7	0.773
		CG	58 43.6	119 42.7		CG	52 39.1	124 44.4		AG	51 38.3	117 41.9	
		GG	64 48.1	128 45.9		CC	10 7.5	22 7.9		AA	7 5.3	15 5.4	
Isolation	5.55	CC	17 12.5	26 9.4	0.575	GG	65 47.8	139 50.4	0.402	GG	71 52.2	151 54.7	0.693
		CG	59 43.4	118 42.7		CG	57 41.9	119 43.1		AG	56 41.2	112 40.6	
		GG	60 44.1	132 47.8		CC	14 10.3	18 6.5		AA	9 6.6	13 4.7	
Projective identification	5.64	CC	11 10.0	32 10.6	0.306	GG	64 58.2	140 46.4	0.105	GG	70 63.6	152 50.3	0.044
		CG	41 37.3	136 45.0		CG	39 35.5	137 45.4		AG	34 30.9	134 44.4	
		GG	58 52.7	134 44.4		CC	7 6.4	25 8.3		AA	6 5.5	16 5.3	
Denial	5.88	CC	16 9.8	27 10.9	0.914	GG	81 49.4	123 49.6	0.785	GG	86 52.4	136 54.8	0.796
		CG	70 42.7	107 43.1		CG	72 43.9	104 41.9		AG	70 42.7	98 39.5	
		GG	78 47.6	114 46.0		CC	11 6.7	21 8.5		AA	8 4.9	14 5.6	
Affiliation	4.07	CC	10 9.3	33 10.9	0.768	GG	53 49.5	151 49.5	0.991	GG	59 55.1	163 53.4	0.930
		CG	55 41.7	122 43.6		CG	50 37.9	126 45.0		AG	48 36.4	120 42.9	
		GG	63 47.7	129 46.1		CC	10 7.6	22 7.9		AA	7 5.3	15 5.4	
Consumption	4.30	CC	8 8.2	35 11.0	0.191	GG	52 54.7	152 47.9	0.490	GG	59 62.1	163 51.4	0.094
		CG	35 36.8	142 44.8		CG	37 38.9	139 43.8		AG	34 35.8	134 42.3	
		GG	52 54.7	140 44.2		CC	6 6.3	26 8.2		AA	2 2.1	20 6.3	
Anticipation	2.95	CC	15 10.6	28 10.4	0.899	GG	75 52.8	129 47.8	0.599	GG	82 57.7	140 51.9	0.471
		CG	63 44.4	114 42.2		CG	56 39.4	120 44.4		AG	54 38.0	114 42.2	
		GG	64 45.1	128 47.4		CC	11 7.7	21 7.8		AA	6 4.2	16 5.9	
**Mature defense**	5.14	CC	12 10.7	31 10.3	0.496	GG	50 44.6	154 51.3	0.274	GG	54 48.2	168 56.0	0.366
		CG	53 47.3	124 41.3		CG	50 44.6	126 42.0		AG	51 45.5	117 39.0	
		GG	47 42.0	145 48.3		CC	12 10.7	20 6.7		AA	7 6.3	15 5.0	
Sublimation	5.38	CC	9 7.1	34 11.9	0.319	GG	55 43.7	149 52.1	0.075	GG	61 48.4	161 56.3	0.253
		CG	58 46.0	119 41.6		CG	64 50.8	112 39.2		AG	59 46.8	109 38.1	
		GG	59 46.8	133 46.5		CC	7 5.6	25 8.7		AA	6 4.8	16 5.6	
Suppression	4.16	CC	13 11.1	30 10.2	0.769	GG	57 48.7	147 49.8	0.737	GG	63 53.8	159 53.9	0.932
		CG	47 40.2	130 44.1		CG	49 41.9	127 43.1		AG	47 40.2	121 41.0	
		GG	57 48.7	135 45.8		CC	11 9.4	21 7.1		AA	7 6.0	15 5.1	
Humor	6.39	CC	15 10.3	28 10.5	0.598	GG	70 48.3	134 50.2	0.918	GG	77 53.1	145 54.3	0.843
		CG	55 41.7	122 43.6		CG	50 37.9	126 45.0		AG	48 36.4	120 42.9	
		GG	63 47.7	129 46.1		CC	10 7.6	22 7.9		AA	7 5.3	15 5.4	

### Comparison of DSQ Scores Between Male and Female Subjects

When the DSQ scores were compared between male and female subjects, **s**ignificant differences were found in 10 defense styles including fantasy, regression, inhibition, withdrawal, omnipotence, isolation, projective identification, sublimation, suppression, and humor (Table 6, all *P* < 0.05). The suppression score for males was higher than that for females, while the scores of other nine defense styles were higher for females.

**Table 6:** Comparison of defense mechanisms between male and female subjects.

**Defense mechanisms**	**Defense score (Mean ± SD)**		***t***	***P***
	**Male**	**Female**		
**Immature defense**	3.65 ± 0.74	3.64 ± 0.76	0.054	0.957
Projection	2.90 ± 0.81	2.76 ± 0.85	1.761	0.079
Passive aggression	3.34 ± 0.97	3.28 ± 1.09	0.554	0.580
Acting out	4.20 ± 1.16	4.29 ± 1.30	−0.751	0.453
Help-rejecting complaining	3.64 ± 1.18	3.69 ± 1.28	−0.412	0.680
Fantasy	3.98 ± 1.95	4.42 ± 2.16	−2.160	0.031
Splitting	4.44 ± 1.12	4.36 ± 1.14	0.681	0.496
Regression	3.85 ± 1.47	4.20 ± 1.66	−2.233	0.026
Somatization	4.72 ± 1.48	4.61 ± 1.47	0.743	0.458
**Intermediate defense**	5.02 ± 0.93	5.19 ± 0.92	−1.958	0.051
Reaction formation	5.59 ± 1.29	5.58 ± 1.25	0.026	0.980
Undoing	5.46 ± 1.29	5.58 ± 1.48	−0.838	0.403
Inhibition	4.14 ± 1.13	4.53 ± 1.13	−3.563	<0.001
Withdrawal	4.27 ± 0.52	4.38 ± 0.56	−2.053	0.041
Idealization	4.05 ± 0.97	4.15 ± 1.02	−0.950	0.343
Pseudo-altruism	4.79 ± 1.09	4.71 ± 1.35	0.701	0.484
Omnipotence	4.64 ± 0.91	5.06 ± 1.03	−4.412	<0.001
Isolation	4.56 ± 1.38	5.06 ± 1.48	−3.590	<0.001
Projective identification	4.56 ± 1.51	5.02 ± 1.78	−2.885	0.004
Denial	4.86 ± 1.92	4.95 ± 1.98	−0.451	0.652
Affiliation	3.69 ± 0.86	3.55 ± 0.93	1.488	0.137
Consumption	3.84 ± 0.80	3.88 ± 0.96	−0.436	0.663
Anticipation	2.27 ± 1.44	2.18 ± 1.50	0.617	0.537
**Mature defense**	4.60 ± 1.00	4.69 ± 0.98	−0.972	0.332
Sublimation	4.45 ± 1.50	4.77 ± 1.54	−2.111	0.035
Suppression	3.81 ± 1.15	3.38 ± 1.11	3.932	<0.001
Humor	5.55 ± 1.47	5.84 ± 1.28	−2.191	0.029

## Discussion

The main finding of the present study was that *NTR1* gene SNPs were significantly associated with two particular defense styles (reaction formation and projective identification) and intermediate defense factors to which these two defense styles belong, in healthy Han-Chinese subjects, and that this association was specific to males. On the part of an association between the *NTR1* gene and DSQ-measured defenses, any relevant research reports were not be found before the present study.

Defense mechanisms have been considered to result from the interaction of biological and environmental factors (20, 26), but the research about the biogenetic basis for defenses has been rarely studied. Generally, defense mechanisms are considered to be an enduring and important dimension of personality and not just an epiphenomena of psychopathology (8). Many previous studies have demonstrated a significant association between personality traits measured by TPQ and alleles encoding dopamine receptors (DRs) and molecules mediating the synthesis, metabolism, and transport of dopamine, for example, functional variants in the *D2DR* (39, 40), the dopamine D4 receptor (*D4DR*) (40–43), the catechol-O-methyltransferase (*COMT*) (42, 44, 45), and the monoamine oxidase A (*MAOA*) genes (44, 46). Direct studies regarding the dopamine biogenetic basis of defenses measured by DSQ have been very rare. Coming et al. demonstrated that subjects with the *DRD2* gene haplotype 1 tended to show a decrease in mature and an increase in neurotic and immature, defense styles compared with those without the haplotype 1, which suggested that the *DRD2* locus was one of the factors controlling defense styles (25). Huang et al. demonstrated that the *PPP1R1B* gene, encoding DARPP-32, was one of the factors responsible for defenses, because *PPP1R1B* polymorphisms were found to be associated with immature defenses (26). Gene variants in the dopamine system might thus be associated with defense mechanisms.

Previous studies have concluded strong evidence for a regulatory role of NT in the dopamine system. Animal and human radioimmunoassay studies have found that NT was present in all the mammalian brain structures containing dopamine nerve cell bodies and terminals (30, 47). Moreover, *NTR1* localized both pre-synaptically and post-synaptically at dopaminergic synapses expressing *DRD2* (48). The anatomical overlap between NT, *NTR1*, dopamine, and *DRD2* suggests possible interactions that regulate neurophysiological functions at the cellular level. Numerous studies have demonstrated that NT could regulate the affinity of DRs for dopamine and dopamine receptor agonists (30, 32, 49–51), in addition to its action on the excitability of dopaminergic neurons (33, 52–54). In the present study, we demonstrated that three *NTR1* gene polymorphisms were significantly associated with projective identification and that the rs6011914 polymorphism was significantly associated with reaction formation in healthy Han-Chinese males. We also demonstrated significant differences in genotype distributions of the rs2427422 polymorphism between the low and high score subgroups for three defense styles: help-rejecting complaining, regression, and projective identification. These results were consistent with and further confirmed the previous study conclusion about the significant association between gene variants in the dopamine system and defense mechanisms (25, 26).

Bond reviewed the published studies about the relationships of defense styles with psychopathology and change and proposed that defense styles should become more adaptive with improvement in symptoms, but intermediate defenses tended to become stable over time (19). Recently, Hayden et al. also demonstrated that maladaptive defense mechanisms were significantly reduced during inpatient therapy and remained low until follow-up, whereas neurotic (intermediate) and adaptive defense mechanisms did not change significantly (5). In other words, intermediate defenses have been found to be a trait-like factor among the three defense factors. In a previous study, our team uncovered a particular defense style (undoing) of intermediate defense, which was demonstrated to be associated with *FYN* gene polymorphisms (55). In the present study, both reaction formation and projective identification, which were demonstrated to be significantly associated with *NTR1* gene polymorphisms, were also classified to be intermediate defenses. Therefore, these results might account, at least partially, for the stability of intermediate defenses, and also indicate that relatively stable biogenetic factors including the dopamine system played an important role in the determination of these intermediate defenses.

Moreover, the present study found significant differences among different genotypes in six particular defense styles and intermediate and mature defenses in males, and in three particular defense styles and intermediate defense in females. Interestingly, after the Bonferroni correction, only the differences in the projective identification score among the rs6090453 genotypes; in the intermediate defense, reaction formation, and projective identification scores among the rs6011914 genotypes; and in the projective identification score among the rs2427422 genotypes were still significant in males. The association between *NTR1* gene polymorphisms and defenses was thus a sex-specific result. In fact, several previous studies have demonstrated significant differences in defenses between the two genders (56–58), and in agreement, we found significant differences in 10 particular defense styles between male and female subjects in the present study. Moreover, the important effect of the interaction between gender and gene polymorphisms has already been demonstrated on individual differences in personality traits (59–61). Therefore, it was expected that our findings were mainly restricted to men. In the present study, we have demonstrated that no significant difference in the frequencies of five haplotypes composed of the alleles in the three *NTR1* SNPs existed between the males and females, indicating no sex-specific difference in the haplotype frequency of *NTR1* SNPs. Therefore, the present sex-specific findings may be the result of androgens, which might be involved in the effect of the *NTR1* gene on neurotransmitter systems, and thus ultimately affect the biological determination of defenses. Biochemical experiments will be needed to determine if this conjecture is indeed true.

## Conclusion

We investigated the associations between three *NTR1* polymorphisms and defense mechanisms measured by DSQ-88 in a large healthy Chinese Han population to demonstrate possible biogenetic mechanisms affecting defenses. We found significant associations in male subjects between three SNPs and the defense style of projective identification as well as the *NTR1* rs6011914 polymorphism and the defense style of reaction formation. Therefore, our results provide evidence that gene variants in the NT system can influence the formation and development of defense mechanisms by regulating the dopamine system and that this effect is affected by gender. However, this conclusion should be considered cautiously because of the limited sample size and number of studied SNPs, and the use of a self-rating scale. Further studies of these *NTR1* SNPs, as well as other SNPs associated with the dopamine system in larger Chinese Han and other ethnic populations are needed to verify our results and to gain a more comprehensive understanding of the effects of *NTR1* allelic variants on defenses.

## Data Availability Statement

The original contributions presented in the study are included in the article/Supplementary Material, further inquiries can be directed to the corresponding author/s.

## Ethics Statement

The studies involving human participants were reviewed and approved by the Ethics Committee of China Medical University. The patients/participants provided their written informed consent to participate in this study.

## Author Contributions

HM and GZ: conceptualization. HM and ML: methodology. HM and JT: software, formal analysis, data curation, and visualization. ML and LZ: validation. HM and LZ: investigation. GZ: resources, supervision, project administration, and funding acquisition. HM and ML: writing—original draft preparation. HM, ML, JT, and GZ: writing—review and editing. All authors contributed to the article and approved the submitted version.

## Funding

This study was supported by a Grant from the Major Project of the Department of Science & Technology of Liaoning Province (2019JH8/10300019).

## Conflict of Interest

The authors declare that the research was conducted in the absence of any commercial or financial relationships that could be construed as a potential conflict of interest. The reviewer AA declared a shared affiliation, with no collaboration, with the authors GZ and ML at the time of the review.

## Publisher's Note

All claims expressed in this article are solely those of the authors and do not necessarily represent those of their affiliated organizations, or those of the publisher, the editors and the reviewers. Any product that may be evaluated in this article, or claim that may be made by its manufacturer, is not guaranteed or endorsed by the publisher.
