# Supplementary material for: Association of Neurotensin Receptor 1 Gene Polymorphisms With Defense Mechanisms in Healthy Chinese
Source: Front Psychiatry. 2021 Nov 17;12:762276. doi: 10.3389/fpsyt.2021.762276 (PMC8635706; doi:10.3389/fpsyt.2021.762276)
Supplement: Supplementary file 1 [file Table_1.DOCX]

Supplementary Table 1 General demographic characteristics of the studied subjects

| Gender | male | female | total | *t* | *p* |
| --- | --- | --- | --- | --- | --- |
| Number | 196(47.6%) | 216(52.4%) | 412(100%) |  |  |
| Age range | 20-58 | 19-57 | 19-58 |  |  |
| Mean age | 31.99±8.13 | 30.93±8.17 | 31.43±8.16 | 1.322 | 0.187 |
