# Supplementary material for: Association of Neurotensin Receptor 1 Gene Polymorphisms With Defense Mechanisms in Healthy Chinese
Source: Front Psychiatry. 2021 Nov 17;12:762276. doi: 10.3389/fpsyt.2021.762276 (PMC8635706; doi:10.3389/fpsyt.2021.762276)
Supplement: Supplementary file 2 [file Table_2.DOCX]

Supplementary Table 2 Genotype frequencies of three *NTR1* gene polymorphisms and the HWE results

| SNP | Genotype | N(%) | χ^2^ | *p* |
| --- | --- | --- | --- | --- |
| rs6090453 | CC | 43(10.4%) | 0.054 | 0.816 |
|  | CG | 177(43.0%) |  |  |
|  | GG | 192(46.6%) |  |  |
| rs6011914 | GG | 204(49.5%) | 0.496 | 0.481 |
|  | CG | 176(42.7%) |  |  |
|  | CC | 32(7.8%) |  |  |
| rs2427422 | GG | 222(53.9%) | 1.847 | 0.174 |
|  | AG | 168(40.8%) |  |  |
|  | AA | 22(5.3%) |  |  |
