# Supplementary material for: Association of Neurotensin Receptor 1 Gene Polymorphisms With Defense Mechanisms in Healthy Chinese
Source: Front Psychiatry. 2021 Nov 17;12:762276. doi: 10.3389/fpsyt.2021.762276 (PMC8635706; doi:10.3389/fpsyt.2021.762276)
Supplement: Supplementary file 3 [file Table_3.DOCX]

Supplementary Table 3 Comparation of genotype distributions of three NTR1 gene polymorphisms between male high- and low-score subgroups for three defense mechanisms

|  | Immature defense | |  | Intermediate defense | |  | Mature defense | |
| --- | --- | --- | --- | --- | --- | --- | --- | --- |
|  | High  n(%) | Low  n(%) |  | High  n(%) | Low  n(%) |  | High  n(%) | Low  n(%) |
| rs6090453 |  |  |  |  |  |  |  |  |
| CC | 3(6.1) | 16(10.9) |  | 2(3.1) | 17(13.0) |  | 5(9.1) | 14(9.9) |
| CG | 26(53.1) | 65(44.2) |  | 33(50.8) | 58(44.3) |  | 30(54.5) | 61(43.3) |
| GG | 20(40.8) | 66(44.9) |  | 30(46.2) | 56(42.7) |  | 20(36.4) | 66(46.8) |
| χ^2^ | 1.618 |  |  | 4.902 |  |  | 2.097 |  |
| *P* | 0.445 |  |  | 0.086 |  |  | 0.350 |  |
| rs6011914 |  |  |  |  |  |  |  |  |
| GG | 23(46.9) | 74(50.3) |  | 32(49.2) | 65(49.6) |  | 23(41.8) | 74(52.5) |
| CG | 24(49.0) | 62(42.2) |  | 32(49.2) | 54(41.2) |  | 27(49.1) | 59(41.8) |
| CC | 2(4.1) | 11(7.5) |  | 1(1.5) | 12(9.2) |  | 5(9.1) | 8(5.7) |
| χ^2^ | 1.115 |  |  | 4.442 |  |  | 2.079 |  |
| *P* | 0.573 |  |  | 0.109 |  |  | 0.354 |  |
| rs2427422 |  |  |  |  |  |  |  |  |
| GG | 26(53.1) | 77(52.4) |  | 35(53.8) | 68(51.9) |  | 26(47.3) | 77(54.6) |
| AG | 22(44.9) | 63(42.9) |  | 29(44.6) | 56(42.7) |  | 28(50.9) | 57(40.4) |
| AA | 1(2.0) | 7(4.8) |  | 1(1.5) | 7(5.3) |  | 1(1.8) | 7(5.0) |
| χ^2^ | 0.705 |  |  | 1.607 |  |  | 2.368 |  |
| *P* | 0.703 |  |  | 0.448 |  |  | 0.306 |  |
