# Supplementary material for: Association of Neurotensin Receptor 1 Gene Polymorphisms With Defense Mechanisms in Healthy Chinese
Source: Front Psychiatry. 2021 Nov 17;12:762276. doi: 10.3389/fpsyt.2021.762276 (PMC8635706; doi:10.3389/fpsyt.2021.762276)
Supplement: Supplementary file 4 [file Table_4.DOCX]

Supplementary Table 4 Comparation of genotype distributions of three NTR1 gene polymorphisms between female high- and low-score subgroups for three defense mechanisms

|  | Immature defense | |  | Intermediate defense | |  | Mature defense | |
| --- | --- | --- | --- | --- | --- | --- | --- | --- |
|  | High  n(%) | Low  n(%) |  | High  n(%) | Low  n(%) |  | High  n(%) | Low  n(%) |
| rs6090453 |  |  |  |  |  |  |  |  |
| CC | 6(9.5) | 18(11.8) |  | 10(14.5) | 14(9.5) |  | 7(12.3) | 17(10.7) |
| CG | 25(39.7) | 61(39.9) |  | 25(35.2) | 61(41.5) |  | 23(40.4) | 63(39.6) |
| GG | 32(50.8) | 74(48.4) |  | 34(49.3) | 72(49.0) |  | 27(47.4) | 79(49.7) |
| χ^2^ | 0.256 |  |  | 1.371 |  |  | 0.147 |  |
| *P* | 0.880 |  |  | 0.504 |  |  | 0.929 |  |
| rs6011914 |  |  |  |  |  |  |  |  |
| GG | 33(52.4) | 74(48.4) |  | 36(52.2) | 71(48.3) |  | 27(47.4) | 80(50.3) |
| CG | 24(38.1) | 66(43.1) |  | 25(36.2) | 65(44.2) |  | 23(40.4) | 67(42.1) |
| CC | 6(9.5) | 13(8.5) |  | 8(11.6) | 11(7.5) |  | 7(12.3) | 12(7.5) |
| χ^2^ | 0.471 |  |  | 1.763 |  |  | 1.174 |  |
| *P* | 0.790 |  |  | 0.414 |  |  | 0.556 |  |
| rs2427422 |  |  |  |  |  |  |  |  |
| GG | 36(57.1) | 83(54.2) |  | 39(56.5) | 80(54.4) |  | 28(49.1) | 91(57.2) |
| AG | 24(38.1) | 59(38.6) |  | 23(33.3) | 60(40.8) |  | 23(40.4) | 60(37.3) |
| AA | 3(4.8) | 11(7.2) |  | 7(10.1) | 7(4.8) |  | 6(10.5) | 8(5.0) |
| χ^2^ | 0.476 |  |  | 2.821 |  |  | 2.530 |  |
| *P* | 0.778 |  |  | 0.244 |  |  | 0.282 |  |
