# Supplementary material for: Association of Neurotensin Receptor 1 Gene Polymorphisms With Defense Mechanisms in Healthy Chinese
Source: Front Psychiatry. 2021 Nov 17;12:762276. doi: 10.3389/fpsyt.2021.762276 (PMC8635706; doi:10.3389/fpsyt.2021.762276)
Supplement: Supplementary file 5 [file Table_5.DOCX]

**防御方式问卷（Defense Style Questionnaire，DSQ）**

**指导语：**本问卷共包括88道题目，请仔细阅读下面的每一道题目，然后根据自己的实际情况认真选择答案。这里不存在正确或错误的答案。每道题目有9个答案，分别用1，2，3，4，5，6，7，8，9来表示：1、完全反对，2、很反对，3、比较反对，4、稍微反对，5、既不反对也不同意，6、稍微同意，7、比较同意，8、很同意，9、完全同意。每道题目只允许选一个答案，请在答题卡相应的位置打钩√，请注意不要漏答。

**INSTRUCTIONS:** This questionnaire consists of a number of statements about personal attitudes. There are no right or wrong answers. Using the 9-point scale shown below, please indicate how much you agree or disagree with each statement by circling one of the numbers on the scale beside the statement. For example, a score of 5 would indicate that you neither agree nor disagree with the statement, a score of 3 that you moderately disagree, a score of 9 that you strongly agree.

**1 2 3 4 5 6 7 8 9**

|  |  |  |  |  |  |  |  |
| --- | --- | --- | --- | --- | --- | --- | --- |

Strongly Strongly

disagree agree

| 序号  No. | 题目  Items | 完全反对 | 很反对 | 比较反对 | 稍微反对 | 既不反对也不同意 | 稍微同意 | 比较同意 | 很同意 | 完全同意 |
| --- | --- | --- | --- | --- | --- | --- | --- | --- | --- | --- |
| 1 | 我从帮助他人而获得满足，如果不这样做，我就会变得情绪抑郁。  I get satisfaction from helping others and if this were taken away from me I would get depressed. | 1 | 2 | 3 | 4 | 5 | 6 | 7 | 8 | 9 |
| 2 | 人们常说我是个脾气暴躁的人。  People often call me a sulker. | 1 | 2 | 3 | 4 | 5 | 6 | 7 | 8 | 9 |
| 3 | 在我没有时间处理某个棘手的事情时，我可以把它搁置一边。  I’m able to keep a problem out of my mind until I have time to deal with it. | 1 | 2 | 3 | 4 | 5 | 6 | 7 | 8 | 9 |
| 4 | 人们总是不公平地对待我。  I’m always treated unfairly. | 1 | 2 | 3 | 4 | 5 | 6 | 7 | 8 | 9 |
| 5 | 我通过做一些积极的或预见性的事情来摆脱自己的焦虑不安，如绘画、做木工活等  I work out my anxiety through doing something constructive and creative like painting or woodwork. | 1 | 2 | 3 | 4 | 5 | 6 | 7 | 8 | 9 |
| 6 | 偶尔，我把一些今天该做的事情推迟到明天做。  Once in a while I put off until tomorrow what I ought to do today. | 1 | 2 | 3 | 4 | 5 | 6 | 7 | 8 | 9 |
| 7 | 我不知道为什么总是遇到相同的受挫情境。  I keep getting into the same type of frustrating situations and I don’t know why. | 1 | 2 | 3 | 4 | 5 | 6 | 7 | 8 | 9 |
| 8 | 我能够相当轻松地嘲笑我自己。  I’m able to laugh at myself pretty easily. | 1 | 2 | 3 | 4 | 5 | 6 | 7 | 8 | 9 |
| 9 | 我受到挫折时，表现就象个孩子。  I act like a child when I’m frustrated. | 1 | 2 | 3 | 4 | 5 | 6 | 7 | 8 | 9 |
| 10 | 在维护我的利益方面，我羞于与人计较。  I’m very shy about standing up for my rights with people. | 1 | 2 | 3 | 4 | 5 | 6 | 7 | 8 | 9 |
| 11 | 我比我认识的人中大多数都强。  I am superior to most people I know. | 1 | 2 | 3 | 4 | 5 | 6 | 7 | 8 | 9 |
| 12 | 人们往往虐待我。  People tend to mistreat me. | 1 | 2 | 3 | 4 | 5 | 6 | 7 | 8 | 9 |
| 13 | 如果某人骗了我或偷了我的钱，我宁愿他得到帮助，而不是受惩罚。  If someone mugged me or stole my money, I’d rather he’d be helped than punished. | 1 | 2 | 3 | 4 | 5 | 6 | 7 | 8 | 9 |
| 14 | 偶尔，我想一些坏得不能说出口的事情。  Once in a while I think of things too bad to talk about. | 1 | 2 | 3 | 4 | 5 | 6 | 7 | 8 | 9 |
| 15 | 偶尔，我因一些下流的笑话而大笑。  Once in a while I laugh at a dirty joke. | 1 | 2 | 3 | 4 | 5 | 6 | 7 | 8 | 9 |
| 16 | 人们说我象一只驼鸟，把自己的头埋入沙中，换句话说，我往往有意忽视一些不愉快的事情。  People say I’m like an ostrich with my head buried in the sand. In other words, I tend to ignore unpleasant facts as if they didn’t exist. | 1 | 2 | 3 | 4 | 5 | 6 | 7 | 8 | 9 |
| 17 | 我常常不能竭尽全力地与人竞争。  I stop myself from going all out in a competition | 1 | 2 | 3 | 4 | 5 | 6 | 7 | 8 | 9 |
| 18 | 我常感到比和我在一起的人强。  I often feel superior to people I’m with. | 1 | 2 | 3 | 4 | 5 | 6 | 7 | 8 | 9 |
| 19 | 某人正在想剥夺我所得到的一切。  Someone is robbing me emotionally of all I’ve got. | 1 | 2 | 3 | 4 | 5 | 6 | 7 | 8 | 9 |
| 20 | 我有时发怒。  I get angry sometimes. | 1 | 2 | 3 | 4 | 5 | 6 | 7 | 8 | 9 |
| 21 | 我时常在某种内在力量的驱使下，不由自主地做出些行为。  I often am driven to act impulsively | 1 | 2 | 3 | 4 | 5 | 6 | 7 | 8 | 9 |
| 22 | 我宁愿饿死而不愿被迫吃饭。  I’d rather starve than be forced to eat. | 1 | 2 | 3 | 4 | 5 | 6 | 7 | 8 | 9 |
| 23 | 我常常故意忽视一些危险，似乎我是个超人。  I ignore danger as if I were Superman. | 1 | 2 | 3 | 4 | 5 | 6 | 7 | 8 | 9 |
| 24 | 我以有贬低别人威望的能力而自豪。  I pride myself on my ability to cut people down to size. | 1 | 2 | 3 | 4 | 5 | 6 | 7 | 8 | 9 |
| 25 | 人们告诉我：我总有被害的感觉。  People tell me I have a persecution complex. | 1 | 2 | 3 | 4 | 5 | 6 | 7 | 8 | 9 |
| 26 | 有时感觉不好时，我就发脾气。  Sometimes when I am not feeling well I am cross. | 1 | 2 | 3 | 4 | 5 | 6 | 7 | 8 | 9 |
| 27 | 当某些事情使我烦恼时，我常常不由自主地做出些行为。  I often act impulsively when something is bothering me. | 1 | 2 | 3 | 4 | 5 | 6 | 7 | 8 | 9 |
| 28 | 当遇事不顺心时，我就会生病。  I get physically ill when things aren’t going well for me. | 1 | 2 | 3 | 4 | 5 | 6 | 7 | 8 | 9 |
| 29 | 我是一个很有自制力的人。  I’m a very inhibited person. | 1 | 2 | 3 | 4 | 5 | 6 | 7 | 8 | 9 |
| 30 | 我简直就像一个不得志的艺术家一样。  I’m a real put-down artist. | 1 | 2 | 3 | 4 | 5 | 6 | 7 | 8 | 9 |
| 31 | 我不总是说真话。  I do not always tell the truth. | 1 | 2 | 3 | 4 | 5 | 6 | 7 | 8 | 9 |
| 32 | 当我感到自尊心受伤害时，我就会回避。  I withdraw from people when I feel hurt. | 1 | 2 | 3 | 4 | 5 | 6 | 7 | 8 | 9 |
| 33 | 我常常不由自主地迫使自己干些过头的事情，以至于其他人不得不限制我。  I often push myself so far that other people have to set limits for me. | 1 | 2 | 3 | 4 | 5 | 6 | 7 | 8 | 9 |
| 34 | 我的朋友们把我看做乡下佬。  My friends see me as a clown. | 1 | 2 | 3 | 4 | 5 | 6 | 7 | 8 | 9 |
| 35 | 在我愤怒的时候，我常常回避。  I withdraw when I’m angry. | 1 | 2 | 3 | 4 | 5 | 6 | 7 | 8 | 9 |
| 36 | 我往往对那些确实对我友好的人，比我应该怀疑的人保持更高的警惕性。  I tend to be on my guard with people who turn out to be more friendly than I would have suspected. | 1 | 2 | 3 | 4 | 5 | 6 | 7 | 8 | 9 |
| 37 | 我已学得特殊的才能，足以使我毫无问题地渡过一生。  I’ve got special talents that allow me to go through life with no problems. | 1 | 2 | 3 | 4 | 5 | 6 | 7 | 8 | 9 |
| 38 | 有时，在选举的时候，我往往选那些我几乎不了解的人。  Sometimes at elections I vote for men about whom I know very little. | 1 | 2 | 3 | 4 | 5 | 6 | 7 | 8 | 9 |
| 39 | 我常常不能按时赴约。  I’m often late for appointments. | 1 | 2 | 3 | 4 | 5 | 6 | 7 | 8 | 9 |
| 40 | 我幻想的多，可在现实生活中做的少。  I work more things out in my daydreams than in my real life. | 1 | 2 | 3 | 4 | 5 | 6 | 7 | 8 | 9 |
| 41 | 我羞于与人打交道。  I’m very shy about approaching people. | 1 | 2 | 3 | 4 | 5 | 6 | 7 | 8 | 9 |
| 42 | 我什么都不怕。  I fear nothing. | 1 | 2 | 3 | 4 | 5 | 6 | 7 | 8 | 9 |
| 43 | 有时我认为我是个天使，有时我认为我是个恶魔。  Sometimes I think I’m an angel and other times I think I’m a devil. | 1 | 2 | 3 | 4 | 5 | 6 | 7 | 8 | 9 |
| 44 | 在比赛时，我只能赢而不能输。  I would rather win than lose in a game. | 1 | 2 | 3 | 4 | 5 | 6 | 7 | 8 | 9 |
| 45 | 在我愤怒的时候，我变得很愿挖苦人。  I get very sarcastic when I’m angry. | 1 | 2 | 3 | 4 | 5 | 6 | 7 | 8 | 9 |
| 46 | 在我自尊心受伤害时，我就公开反击。  I get openly aggressive when I feel hurt. | 1 | 2 | 3 | 4 | 5 | 6 | 7 | 8 | 9 |
| 47 | 我认为当我受伤害时，我就应该翻脸。  I believe in turning the other cheek when someone hurts me. | 1 | 2 | 3 | 4 | 5 | 6 | 7 | 8 | 9 |
| 48 | 我每天读报时，不是每个版面都读。  I do not read every editorial in the newspaper every day. | 1 | 2 | 3 | 4 | 5 | 6 | 7 | 8 | 9 |
| 49 | 我沮丧时，就会避开。  I withdraw when I’m sad. | 1 | 2 | 3 | 4 | 5 | 6 | 7 | 8 | 9 |
| 50 | 我对性问题感到害羞。  I’m shy about sex. | 1 | 2 | 3 | 4 | 5 | 6 | 7 | 8 | 9 |
| 51 | 我总是感到我所认识的某个人象个保护神。  I always feel that someone I know is like a guardian angel. | 1 | 2 | 3 | 4 | 5 | 6 | 7 | 8 | 9 |
| 52 | 我的处世哲学是：“非理勿信，非理勿做，非理勿视”。  My philosophy is “Hear no evil, do no evil, see no evil.” | 1 | 2 | 3 | 4 | 5 | 6 | 7 | 8 | 9 |
| 53 | 我认为：人有好坏之分。  As far as I’m concerned, people are either good or bad. | 1 | 2 | 3 | 4 | 5 | 6 | 7 | 8 | 9 |
| 54 | 如果我的上司惹我生气，我可能会在工作中找麻烦或磨洋工，以报复他。  If my boss bugged me, I might make a mistake in my work or work more slowly so as to get back at him. | 1 | 2 | 3 | 4 | 5 | 6 | 7 | 8 | 9 |
| 55 | 每个人都和我对着干。  Everyone is against me. | 1 | 2 | 3 | 4 | 5 | 6 | 7 | 8 | 9 |
| 56 | 我往往对那些我讨厌的人表示友好。  I try to be nice to people I don’t like. | 1 | 2 | 3 | 4 | 5 | 6 | 7 | 8 | 9 |
| 57 | 如果我乘坐的飞机的一个发动机失灵，我就会非常紧张。  I would be very nervous if an airplane in which I was flying lost an engine. | 1 | 2 | 3 | 4 | 5 | 6 | 7 | 8 | 9 |
| 58 | 我认识这样一个人，他什么都能做而且做得合理正直。  There is someone I know who can do anything and who is absolutely fair and just. | 1 | 2 | 3 | 4 | 5 | 6 | 7 | 8 | 9 |
| 59 | 如果我感情的发泄会防碍我正从事的事业，那么我就能控制住它。  I can keep the lid on my feelings if it would interfere with what I’m doing if I were to let them out. | 1 | 2 | 3 | 4 | 5 | 6 | 7 | 8 | 9 |
| 60 | 一些人正在密谋要害我。  Some people are plotting to kill me. | 1 | 2 | 3 | 4 | 5 | 6 | 7 | 8 | 9 |
| 61 | 我通常可以看到恶境当中好的一面。  I’m usually able to see the funny side of an otherwise painful predicament. | 1 | 2 | 3 | 4 | 5 | 6 | 7 | 8 | 9 |
| 62 | 在我不得不去做一些我不愿做的事情时，就头痛。  I get a headache when I have to do something I don’t like. | 1 | 2 | 3 | 4 | 5 | 6 | 7 | 8 | 9 |
| 63 | 我常常发现我对那些理应仇视的人，表示很友好。  I often find myself being very nice to people who I should be angry at by all rights. | 1 | 2 | 3 | 4 | 5 | 6 | 7 | 8 | 9 |
| 64 | 我认为：“人人都有善意”是不存在的，如果你不好，那么你一切都不好。  There’s no such thing as “finding a little good in everyone.” If you’re bad, you’re all bad. | 1 | 2 | 3 | 4 | 5 | 6 | 7 | 8 | 9 |
| 65 | 我决不会对那些我讨厌的人表示愤怒。  I should never get angry at people I don’t like. | 1 | 2 | 3 | 4 | 5 | 6 | 7 | 8 | 9 |
| 66 | 我确信生活对我是不公正的。  I am sure I get a raw deal from life. | 1 | 2 | 3 | 4 | 5 | 6 | 7 | 8 | 9 |
| 67 | 在严重的打击下，我就会垮下来。  I fall apart under stress. | 1 | 2 | 3 | 4 | 5 | 6 | 7 | 8 | 9 |
| 68 | 在我意识到不得不面临一场困境的时候，如考试、招工会谈，我就试图想像它会如何，并计划出一些方法去应付它。  When I know that I will have to face a difficult situation like an exam or a job interview，I try to image what it will be like and plan ways to cope with it. | 1 | 2 | 3 | 4 | 5 | 6 | 7 | 8 | 9 |
| 69 | 医生们决不会真的弄清我患的是什么病。  Doctors never really understand what is wrong with me. | 1 | 2 | 3 | 4 | 5 | 6 | 7 | 8 | 9 |
| 70 | 当某个和我很亲近的人死去时，我并不悲伤。  When someone close to me dies, I don’t feel upset. | 1 | 2 | 3 | 4 | 5 | 6 | 7 | 8 | 9 |
| 71 | 在我为了利益和人争斗之后，我往往因为我的粗鲁而向人道歉。  After I fight for my rights, I tend to apologize for my assertiveness. | 1 | 2 | 3 | 4 | 5 | 6 | 7 | 8 | 9 |
| 72 | 发生与我有关的大部分事情并不是我的责任。  Most of what happens to me is not my responsibility. | 1 | 2 | 3 | 4 | 5 | 6 | 7 | 8 | 9 |
| 73 | 当我感觉情绪压抑或焦虑不安时，吃点东西，可以使我感觉好些。  When I’m depressed or anxious, eating makes me feel better. | 1 | 2 | 3 | 4 | 5 | 6 | 7 | 8 | 9 |
| 74 | 勤奋工作使我感觉好些。  Hard work makes me feel better. | 1 | 2 | 3 | 4 | 5 | 6 | 7 | 8 | 9 |
| 75 | 医生不能真的帮我解决问题。  Doctors are not able to help me really get over my problems. | 1 | 2 | 3 | 4 | 5 | 6 | 7 | 8 | 9 |
| 76 | 我常听人们说我不暴露自己的感情。  I’m often told that I don’t show my feelings. | 1 | 2 | 3 | 4 | 5 | 6 | 7 | 8 | 9 |
| 77 | 我认为，人们在看电影，戏剧或书籍时所领悟的意义，比这些作品所要表达的意义要多。  I believe that people usually see more meaning in films, plays, or books than is actually there. | 1 | 2 | 3 | 4 | 5 | 6 | 7 | 8 | 9 |
| 78 | 我感觉到我有一些不由自主要去做的习惯或仪式行为，给我带来很多麻烦。  I have habits or rituals which I feel compelled to do or else something terrible will happen. | 1 | 2 | 3 | 4 | 5 | 6 | 7 | 8 | 9 |
| 79 | 当我紧张时，就喝酒或吃药。  I take drugs, medicine or alcohol when I’m tense. | 1 | 2 | 3 | 4 | 5 | 6 | 7 | 8 | 9 |
| 80 | 当我心情不愉快时，就想和别人呆在一起。  When I feel bad, I try to be with someone. | 1 | 2 | 3 | 4 | 5 | 6 | 7 | 8 | 9 |
| 81 | 如果我能够预感到我会沮丧的话，我就能更好地应付它。  If I can predict that I’m going to be sad ahead of time, I can cope better. | 1 | 2 | 3 | 4 | 5 | 6 | 7 | 8 | 9 |
| 82 | 无论我怎样发牢骚，从未得到过满意的结果。  No matter how much I complain, I never get a satisfactory response | 1 | 2 | 3 | 4 | 5 | 6 | 7 | 8 | 9 |
| 83 | 我常常发现当环境要引起我强烈的情绪反应时，我就会麻木不仁。  Often I find that I don’t feel anything when the situation would seem to warrant strong emotions. | 1 | 2 | 3 | 4 | 5 | 6 | 7 | 8 | 9 |
| 84 | 忘我地工作，可使我摆脱情绪上的忧郁和焦虑。  Sticking to the task at hand keeps me from feeling depressed or anxious. | 1 | 2 | 3 | 4 | 5 | 6 | 7 | 8 | 9 |
| 85 | 紧张的时候，我就吸烟。  I smoke when I’m nervous. | 1 | 2 | 3 | 4 | 5 | 6 | 7 | 8 | 9 |
| 86 | 如果我陷入某种危机时，我就会寻找另一个和我具有同样命运的人。  If I were in a crisis, I would seek out another person who had the same problem. | 1 | 2 | 3 | 4 | 5 | 6 | 7 | 8 | 9 |
| 87 | 如果我做错了事情，不能受责备。  I cannot be blamed for what I do wrong. | 1 | 2 | 3 | 4 | 5 | 6 | 7 | 8 | 9 |
| 88 | 如果我有攻击他人的想法，我就感觉有种做点事情的需要，以转移这种想法。  If I have an aggressive thought, I feel the need to do something to compensate for it. | 1 | 2 | 3 | 4 | 5 | 6 | 7 | 8 | 9 |
