# Supplementary material for: Association of Neurotensin Receptor 1 Gene Polymorphisms With Defense Mechanisms in Healthy Chinese
Source: Front Psychiatry. 2021 Nov 17;12:762276. doi: 10.3389/fpsyt.2021.762276 (PMC8635706; doi:10.3389/fpsyt.2021.762276)
Supplement: Supplementary file 6 [file Table_6.DOCX]

**健康成年人心理防御机制、人格特征、应对方式、焦虑水平与NTR1、GPx-1、CK1基因多态性的相关性研究**

**知情同意书**

**第一部分 知情告知**

**尊敬的 先生/女士：**

我们诚挚邀请您参加健康成年人心理防御机制、人格特征、应对方式、焦虑水平与NTR1、GPx-1、CK1基因多态性的相关性研究。本研究遵从赫尔辛基宣言原则，已通过中国医科大学伦理委员会审查（伦理批件号：2019-209-2），符合医疗道德。本研究以中国医科大学附属第一医院为研究单位，进行临床研究。

在您决定是否参加这项研究之前，请尽可能仔细阅读以下内容，它可以帮助您了解该项研究以及为何要进行这项研究，研究的程序和期限，参加研究后可能给您带来的益处、风险和不适。您也可以和您的亲属、朋友一起讨论后做出决定。如有任何疑问，请您的医生给予解释。

1. **研究背景和目的**

1、心理防御机制、人格特征与精神障碍和异常情绪行为的发生显著相关，然而其形成的确切机制尚不清楚。前人关于心理防御机制和人格特征的研究，大多集中于心理社会因素的角度，少有生物学、尤其是遗传因素的研究。前人已发表的一些研究结果，间接地提示，NTR1、GPx-1、CK1基因多态性可能与心理防御机制、人格特征关系密切。因此，本研究计划采用心理防御机制问卷（DSQ-88）和三维人格问卷（TPQ）对健康成年汉族人的心理防御机制、人格特征数据进行测量，利用聚合酶链式反应－限制性片段长度多态性（PCR-RFLP）技术对健康成年汉族人的NTR1、GPx-1、CK1基因多态性进行分析，旨在探究上述基因多态性与心理防御机制、人格特征的关系。

2、焦虑是一个从正常的生理性焦虑到异常的病理性焦虑的连续谱，也就是说，焦虑通常是人类一种正常的情绪反应，但是过度的焦虑会导致焦虑性障碍，后者是所有精神疾病中发生率最高的类型。焦虑的产生原因是复杂的、多方面，前人已发表的一些研究结果，间接地提示，防御机制、人格特征、应对方式可能与焦虑水平显著相关；NTR1、GPx-1、CK1基因多态性可能在上述相关路径中发挥调节作用。因此，本研究计划采用心理防御机制问卷（DSQ-88）、三维人格问卷（TPQ）、简易应对方式问卷（CSCQ）和状态-特质焦虑问卷（STAI）对健康成年汉族人的心理防御机制、人格特征、应对方式以及焦虑水平的数据进行测量，利用聚合酶链式反应－限制性片段长度多态性（PCR-RFLP）技术对健康成年汉族人的NTR1、GPx-1、CK1基因多态性进行分析，旨在探究防御机制、人格特征、应对方式以及上述基因多态性在焦虑发生中的作用以及路径。

1. **研究内容和步骤**

**（一）入选标准**

1.汉族；

2.年龄在18-60岁之间；

3.无神经、精神或慢性躯体疾病；

4.两系三代亲属中只允许有1人入选；

5.家属及本人理解研究内容，能够配合完成问卷调查和血液标本采集；

6.患者已签署知情同意。

如果您不符合任意上述条件，您将不能作为受试者入选。

**（二）排除标准**

1.现患有或曾经患者神经、精神疾病；

2.现患有慢性躯体疾病；

3.物质依赖及酒精、物质滥用；

4. 两系三代亲属中已经有人入选本研究。

如果以上任何一个答案为“是”，则此受试者不能参加本研究。

**（三）研究中受试者参与的时间**

入组当天被试者需要花费1.5-2小时的时间完成心测和血液样本采集，后续无操作。

**三、研究中需要您配合完成的事项**

在仔细阅读了本知情同意书及询问了相关问题后，如果您愿意参加本研究，您需要签署一份书面的知情同意书。在您决定参加本研究后，具体您将接受以下措施：

1.**一般信息收集：**基本信息（姓名，性别，年龄，文化程度等）、既往史、家族史等。

2.**心理测验量表的检测：**心理防御机制问卷（DSQ-88）、三维人格问卷（TPQ）、简易应对方式问卷（CSCQ）和状态-特质焦虑问卷（STAI）**。**

3**.血液样本的采集：**收集2ML外周静脉血样本进行基因多态性分析，剩余的标本会按照废弃标本的规定进行处理。

**四、研究费用**

心理防御机制问卷、三维人格问卷、简易应对方式问卷和状态-特质焦虑问卷测验以及外周静脉血的采集，均在中国医科大学附属第一医院精神心理科进行，费用由研究者承担。

**五、参加研究可能的受益与补偿**

**1、研究对受试者本人的受益。**免费的心理防御机制问卷、三维人格问卷、简易应对方式问卷和状态-特质焦虑问卷的检测。心测结果可以帮助您了解自己的人格特征、应对压力时常用的应对方式和防御机制以及目前的焦虑情绪水平等。

**2、研究对社会群体的收益：**从您参与的本研究中得到的信息在将来能够使您和社会大众获益，例如，从探索出的焦虑发生原因和路径入手，降低相关人群的焦虑症状，对推动精神心理问题的多角度综合治疗具有重大意义。

**3、补偿：**参与本次研究，您不会得到报酬。

**六、参加研究可能的风险、不适及处理办法**

您参加的是非干预、观察性研究，心测量表的检测不会对您造成任何不良的影响，外周血采集除了可能会使您有片刻疼痛感外，也不会对您造成任何不良的影响；这些事项需占用您的一些宝贵时间（1.5-2小时）。

**七、自愿参加/退出研究**

研究者邀请您参加本研究，您在研究的任何阶段均有权随时退出而不需申明任何理由，并且不会遭到任何歧视或报复，不需承担之前检测的费用，医疗待遇与权益不受影响。

**八、个人信息的保密**

在研究期间收集到得所有信息都将是保密的，并由研究者保管。只有研究人员与伦理委员会成员及相关部门在法律允许的范围内，有权审阅您的信息记录。在任何有关项目的研究报告和出版物中，您的个人信息不会被独立公开。

**九、联系方式**

您可以在任何时间提出有关本研究的任何问题，或有任何的困扰，请随时与您的医生取得联系。

医生： 联系方式：

如您对参加本研究有任何的抱怨，请联系伦理委员会。

中国医科大学伦理委员会通讯地址：辽宁省沈阳市和平区南京北街155号，邮编110001，办公电话：024-83282837。

感谢您阅读以上材料。如果您决定参加本项研究，请告诉您的医生，他会为您安排一切有关研究的事项。

**健康成年人心理防御机制、人格特征、应对方式、焦虑水平与NTR1、GPx-1、CK1基因多态性的相关性研究**

**知情同意书**

**第二部分 同意签字**

**同意声明：**

1、我已经阅读了上述有关本研究的介绍，并且研究者向我作了详细的解释和说明，我提出的所有问题都得到了满意的答复。

2、我知晓参加研究是自愿的，我确认已有充足时间对此进行考虑，而且明白：

1）我可以随时向医生咨询更多的信息。；

2）我可以随时退出研究，而不受到歧视和报复，医疗权益和待遇不会受到影响。

3、我同意伦理委员会及相关部门查阅我的研究资料。

4、我将获得一份经过签名并注明日期的知情同意书副本。

最后，我决定同意参加本项研究，并愿意按照研究方案要求，与医生配合完成本研究。

受试者签名： 联系电话： 日期： 年 月 日

**研究者声明：**

我确认已向该受试者充分解释和说明了本研究的目的、操作过程以及受试者参加该项目可能存在的风险和利益，满意地回答了该受试者的所有有关问题，并给其一份经过双方签署过的知情同意书副本。

研究者签名： 联系电话： 日期： 年 月 日
